# Tick findings from subterranean environments in the Central German Uplands and Luxembourg reveal a predominance of male *Ixodes hexagonus*

**DOI:** 10.1007/s10493-023-00795-2

**Published:** 2023-04-28

**Authors:** Alexander Weigand, Stefan Zaenker, Dieter Weber, Sabine Schaper, Michael Bröker, Christian Zaenker, Lidia Chitimia-Dobler

**Affiliations:** 1 National Museum of Natural History Luxembourg, 25 Rue Münster, 2160 Luxembourg, Luxembourg; 2 Fondation Faune-Flore, Musée National d’Histoire Naturelle, 25 Rue Münster, 2160 Luxembourg, Luxembourg; 3 Hesse Federation for Cave and Karst Research, Königswarter Str. 2a, 36039 Fulda, Germany; 4 Bundeswehr Institute of Microbiology, Neuherbergstrasse 11, 80937 Munich, Germany; 5 Pappelweg 30, 35041 Marburg, Germany

**Keywords:** Tick-borne diseases, Subterranean biology, Acari, Ixodidae, Underground environments

## Abstract

Questing ticks are usually collected by flagging or dragging. Mostly exophilic tick species are caught, such as *Ixodes ricinus*, the most common tick in Central Europe. In the present study, ticks collected from underground environments in the Grand Duchy of Luxembourg and in the Central German Uplands (Federal States of Hesse, Bavaria, Thuringia, Baden-Wuerttemberg, Rhineland-Palatinate, Saarland and Northrhine-Westphalia) were investigated. Six tick species were revealed among the 396 analyzed specimens: *Ixodes ariadnae*, *Ixodes canisuga*, *Ixodes hexagonus*, *I. ricinus*, *Ixodes trianguliceps*, and *Dermacentor marginatus*. Adults and immatures of *I. hexagonus* dominated the findings (57% of all specimens), especially in shelters acting as potential resting places of main hosts. *Ixodes canisuga* and *I. trianguliceps* were for the first time recorded in Luxembourg, and one nymph of the bat tick *I. ariadnae* represents only the second report for Germany. Collecting ticks in subterranean environments turned out to be a useful approach to increase knowledge about the occurrence of relatively rare tick species, including those that spend most of their lifetime on their hosts, but detach in such environmental settings.

**Supplementary Information:**

The online version contains supplementary material available at 10.1007/s10493-023-00795-2.

## Introduction

Underground environments such as natural caves, abandoned mines and tunnels are often neglected sources of human and animal pathogens and the habitats of their respective arthropod vectors (Montagna et al. 2003; Jurado et al. 2010; Igreja 2011; Obame-Nkoghe et al. 2017). In Central Europe, underground environments harbor important arthropod vector groups, such as mosquitos (Diptera: Culicidae), fleas (Siphonaptera), and ticks (Acari: Ixodidae) (e.g., Zaenker et al. 2020), but taxonomically targeted and geographically more widespread studies in these environments are scarce (Kutzscher and Weber 2015; Dörge et al. 2020; Zittra et al. 2019, 2021; Zaenker et al. 2020).

Four ixodid tick species are currently listed for the Grand Duchy of Luxembourg, i.e., *Ixodes ricinus*, *Ixodes frontalis*, *Ixodes hexagonus*, and *Dermacentor reticulatus* (Reye et al. 2010; Reye 2011; Weigand et al. 2020). Additionally, *Hyalomma marginatum* was recently reported, but this species is considered a rare introduction without established populations in Luxembourg (Weigand et al. 2020). Population densities of *D. reticulatus* have significantly increased in the country in recent years, a phenomenon that can be attributed to an ongoing range expansion of this species in Central Europe (Rubel et al. 2016; Drehmann et al. 2020; Weigand et al. 2020). The only systematic national survey of ticks (2007–2009; >9,700 total specimens) revealed *I. ricinus* as the by far most widespread and common species in Luxembourg (Reye 2011). *Ixodes hexagonus* was occasionally collected from infested animals (predominantly hedgehogs, cats, and foxes), whereas *I. frontalis* was only rarely encountered on birds (Reye 2011).

Tick research in Germany is well established, and much more comprehensive data are available compared to Luxembourg. The German tick fauna currently includes 21 species, 19 ixodid species belonging to three genera: *Dermacentor*, *Haemaphysalis*, and *Ixodes*; and two soft tick species (Petney et al. 2012, 2015; Hornok et al. 2015a; Bröker et al. 2019; Hauck et al. 2020; Ott et al. 2020; Rubel et al. 2021). In addition, two ixodid species, *H. marginatum* and *Hyalomma rufipes*, are often imported into Germany by migratory birds (Chitimia-Dobler et al. 2019), whereas the cosmopolitan *Rhipicephalus sanguineus* enters the country with dogs (Petney et al. 2012). Ticks are also imported into Germany by humans returning from holidays abroad, e.g., *Amblyomma mixtum* from Cuba (Chitimia-Dobler et al. 2020), *Dermacentor auratus* from Cambodia (Chitimia-Dobler et al. 2021), and *Rhipicephalus maculatus* from South Africa (Chitimia-Dobler and Mans 2022).

Central European ticks are known from a variety of habitats (Mierzejewska et al. 2017), e.g., host nests in the case of *Ixodes arboricola* (Heylen et al. 2014a, b). However, most studies of German ticks have been based on flagging or dragging collection methods, especially investigations of *I. ricinus* abundance and/or activity, whether or not such studies focus on tick-borne pathogens (Bröker et al. 2019; Hauck et al. 2020; Ott et al. 2020). Similar techniques have been adopted for investigations involving *Ixodes inopinatus*, *I. frontalis*, *I. hexagonus* and *D. reticulatus*. In the special case of underground environments, four bat-infesting tick species have been reported: *Carios vespertilionis*, *Ixodes simplex*, *Ixodes ariadnae*, and *Ixodes vespertilionis*, in addition to several other non-ixodid species of parasitic or predatory mites (Zaenker et al. 2020). The tick *Ixodes barbarossae*, described by Schulze (1937) from the Kyffhäuserhöhle (Kyffhaeuser cave; Thuringia, Germany), is now considered a junior synonym of *Ixodes canisuga*. In the vast majority of cases, data on underground findings of tick species originate from focused ectoparasitic studies on bats (e.g., Schmidt 1987; Walter 1992; Rupp et al. 2004; Scheffler 2009). For Luxembourg, Weber (2013) conducted a comprehensive national survey of the cave-dwelling fauna, but no data so far exist on ticks collected from underground environments.

This contribution is a retrospective analysis of data on ticks collected in underground environments. Our results will contribute to the knowledge of the tick fauna of underground environments in the Central German Uplands of western Germany (mainly in the Federal States of Hesse, Rhineland-Palatinate, and Saarland) and Luxembourg. Tick findings from long-term biological assessments of subterranean environments are analyzed, and, for Germany, compared with the tick fauna of springs.

## Materials and methods

### Governmental permission and control

According to the German Federal Law on Nature Protection § 30, natural caves, semi-natural mining galleries and spring areas are protected biotopes in Germany. To conduct research in these biotopes and collect animals, the Regional Association for Research on Caves and Karst obtained permission from the Hesse Regional Authorities for Nature Protection, Environment and Geology. Data are reported yearly to the latter office and are archived in the Biospeleological Register (Reiss et al. 2009). Dieter Weber obtained permission from the Struktur- und Genehmigungsdirektion Süd for the Federal State of Rhineland-Palatinate and from the Landesamt für Umwelt- und Arbeitsschutz, Geschäftsbereich 3, Natur- und Umweltschutz for the Federal State of Saarland.

For Luxembourg, tick specimens originated from the study of Weber (2013) and subsequent visits to subterranean sites. Sampling was permitted by the respective owners of subterranean sites or due to the close collaboration of the National Museum of Natural History Luxembourg (MNHNL) with the Ministère de l’Environnement, du Climat et du Développement durable (MECDD) Luxembourg. Specimens are stored in the wet collection of the MNHNL (reference numbers MNHNL67125, MNHNL130068-MNHNL130307).

### Datasets

Subterranean environments are frequently visited for bioassessment of cave-dwelling organisms (Weber 2013; Zaenker et al. 2020), e.g., in the context of monitoring habitat type 8310 (non-touristic caves) of the EU Habitats Directive (Council Directive 92/43/EEC; Weigand et al. 2022). Ticks are routinely observed, but have never been systematically evaluated. Here, tick findings from visits to subterranean sites (natural caves, mines, cellars, tunnels, etc.) in the Central German Uplands (mainly in the Federal States of Hesse, Rhineland-Palatinate and Saarland) and Luxembourg are analyzed. Ticks were not removed from their hosts (e.g., bats), but were directly collected from the environment or detected in Barber traps. Most collecting focused on microenvironments, such as the walls and ceilings of underground sites. Ticks were collected using fine forceps and placed in 70% ethanol in 1.5-mL Eppendorf tubes. In semi-aquatic biotopes, materials such as wood and stones were shaken over a white cup. At some localities, tick specimens were retrieved from Barber traps with 70% isopropanol used as a fixative and preservative.

Available tick findings from regular spring monitoring in the Central German Uplands were included for comparison. Focus was laid on the Rhön mountain region in eastern Hesse and the neighboring Federal States of Bavaria and Thuringia. Most specimens were collected from plants (net catch in spring vegetation), stones and rocks as well as organic and inorganic substrates such as mud, leaves and decaying wood in these aquatic biotopes.

### Specimen identification

Ticks were examined with a Keyence VHX-900F microscope (Itasca, IL, USA) and identified to species level using morphological keys (Filippova 1977; Pérez-Eid 2007; Hornok et al. 2014). In cases of questionable specimens, those were objected to molecular analysis. DNA was extracted using the QIAamp mini DNA extraction kit (Qiagen, Hilden, Germany), according to the manufacturer’s instructions. The 16S rRNA gene was amplified by PCR and Sanger sequenced according to Halos et al. (2004), using the tick-specific primer pair TQ16S + 1F (5’-CTGCTCAATGATTTTTTAAATTGCTGTGG-3’) and TQ16S-2R (5’-ACGCTGTTATCCCTAGAG-3’) of Black and Piesman (1994). Sequences were edited and primers trimmed using the Geneious Prime 2022.2.1 software (https://www.geneious.com). A BLASTN search (Zhang et al. 2000) in GenBank was performed to molecularly annotate the individual 16S rDNA sequences. 16S rDNA sequence data can be found in GenBank and the supplementary material.

## Results

### Germany

**Fig. 1:**
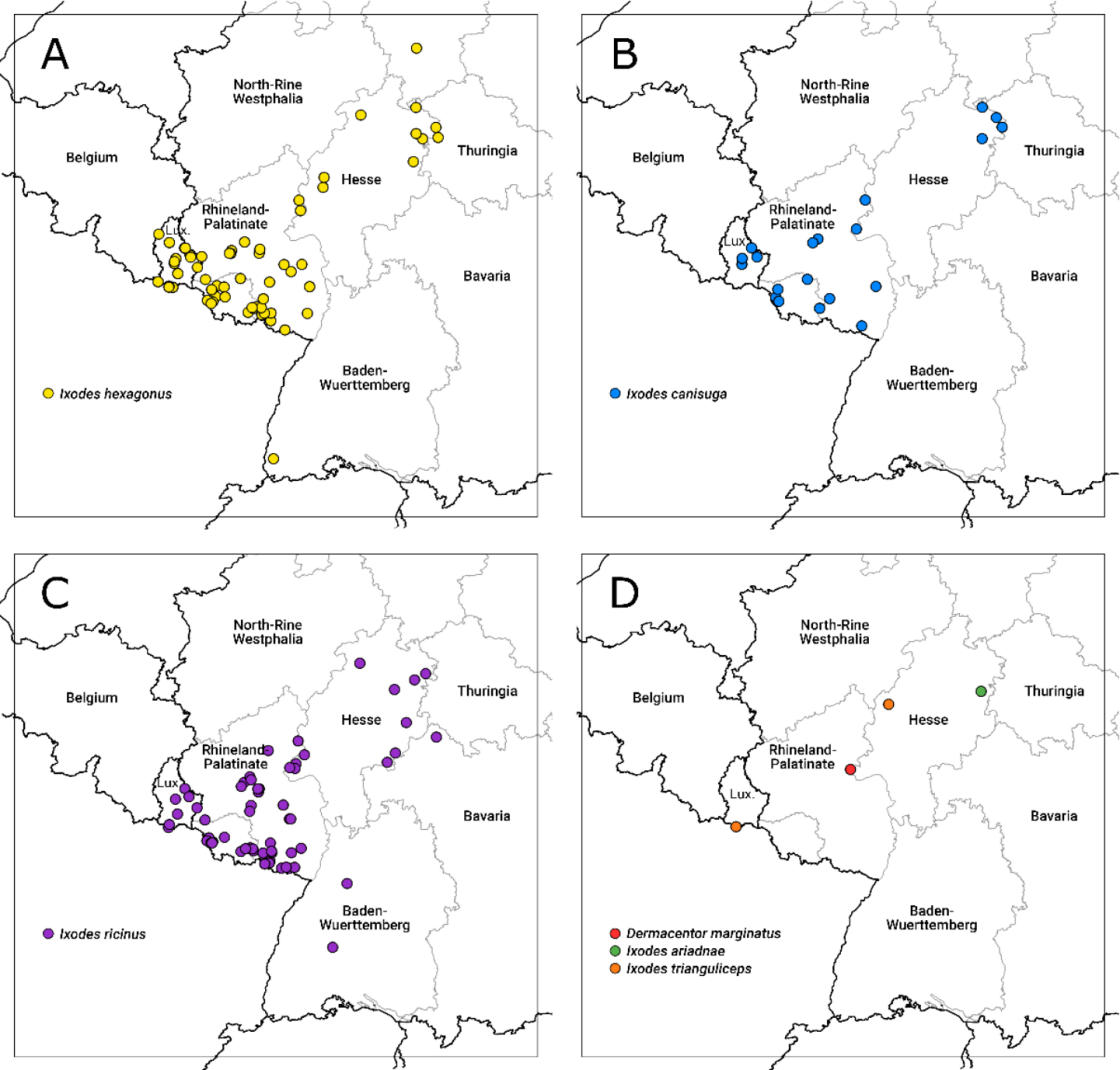
Distribution of tick species detected in subterranean environments of the Central German Uplands and Luxembourg. A: *Ixodes hexagonus*, B: *Ixodes canisuga*, C: *Ixodes ricinus*, D: *Dermacentor marginatus*, *Ixodes ariadnae*, *Ixodes trianguliceps*

**Fig. 2:**
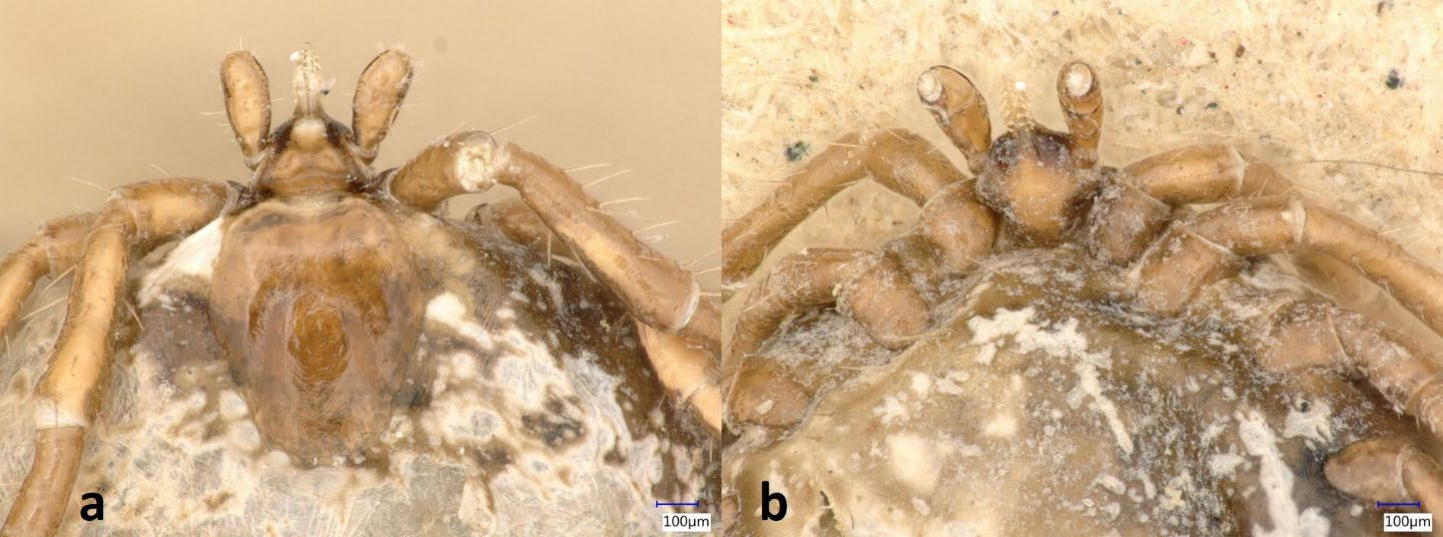
*Ixodes ariadnae* nymph, reference code Mi5737 of the Biospeleological Register of Hesse (Germany). A: dorsal view; B: ventral view

In total, 224 ixodid ticks were collected between August 1997 and January 2022 from 114 subterranean sites in the Central German Uplands, primarily the Federal States of Hesse, Rhineland-Palatinate and Saarland (Table 1). The most frequently observed species were *Ixodes hexagonus* (n = 83; 38 males, 30 females, 14 nymphs, 1 larva) and *I. ricinus* (n = 77; 10 males, 6 females, 39 nymphs, 22 larvae), followed by *I. canisuga* (n = 61; 22 males, 30 females, 4 nymphs, 5 larvae) (Tables 1 and 2, S1 and S2, Fig. 1). Single specimens of *I. ariadnae* (1 nymph), *I. trianguliceps* (1 female), and *Dermacentor marginatus* (1 male) were also recorded. The single *I. ariadnae* nymph was collected in 2021 on the wall of a water passage in Friedewald (Hesse) (Fig. 2). The analysis of the 16S rDNA marker of this specimen verified the initial morphological identification of *I. ariadnae* (GenBank acc. nr. OQ615884; Table S2). The *I. trianguliceps* female was found in 2003, on the wall of a mining tunnel in Dillenburg (Hesse). The *D. marginatus* male was found in 2012, also on the wall of a mining tunnel in Kaub (Rhineland-Palatinate). The tick findings of the spring monitoring data from the Rhön mountain region consisted only of *I. ricinus* specimens in all developmental stages (n = 290; Table S3).

**Table 1:** Overview of tick species in underground environments in Germany (DE) and Luxembourg (LU). Also shown are the ixodid ticks encountered in the frame of the German spring monitoring in the Rhön mountains

Species	No. specimens		Underground habitat type								Spring
			Natural				Artificial				
			Total	DE	LU		Total	DE	LU		DE
*Ixodes ariadnae*	1						1	1			
*Ixodes canisuga*	71		49	39	10		22	22			
*Ixodes hexagonus*	237		90	18	72		147	65	82		
*Ixodes ricinus*	374		20	16	4		64	61	3		290
*Ixodes trianguliceps*	2						2	1	1		
*Dermacentor marginatus*	1						1	1			

**Table 2:** Overview of gender proportions and developmental stages of ixodid ticks found in underground environments in Germany (DE) and Luxembourg (LU).

Species		No. specimens	Males				Females				Nymphs					Larvae		
			Total	DE	LU		Total	DE	LU		Total	DE	LU			Total	DE	LU
*Ixodes ariadnae*		1									1	1						
*Ixodes canisuga*		71	25	22	3		36	30	6		4	4	0			6	5	1
*Ixodes hexagonus*		237	102	38	64		74	30	44		51	14	37			10	1	9
*Ixodes ricinus*		84	12	10	2		7	6	1		40	39	1			25	22	3
*Ixodes trianguliceps*		2					2	1	1									
*Dermacentor marginatus*		1	1	1														

### Luxembourg

In total, 172 ixodid ticks were obtained from 27 underground sites in Luxembourg visited between January 1996 and June 2014 (Table 1). The records were dominated by *I. hexagonus* (n = 154; 64 males, 44 females, 37 nymphs and 9 larvae), followed by *I. canisuga* (n = 10; 3 males, 6 females, 1 larva), *I. ricinus* (n = 7; 2 males, 1 female, 1 nymph, 3 larvae) and *I. trianguliceps* (1 female) (Fig. 1; Tables 1 and 2, S1 and S2). However, more than one-third of all *I. hexagonus* specimens were found at a single site (n = 57, Méischtrefer Hiel), predominantly resulting from three sampling events in winter 2010/2011 (90%). Findings of *I. canisuga* and *I. trianguliceps* represent the first records for this country. The single *I. trianguliceps* female was found in 2007 in the entrance area of Minière Hutbierg. *Ixodes canisuga* records originated from five caves: Fusselach, Bitzmaschinn (both part of the Mamerlayen), Grande Fleur, Déiwepetz and the Méischtrefer Hiel. Records usually were made in the entrance area, but all three findings in the Méischtrefer Hiel stem from the dark zone.

## Discussion

### New and rare faunistic findings

In total, six tick species were detected in the investigated underground environments (*I. ariadnae*, *I. canisuga*, *I. hexagonus*, *I. ricinus*, *I. trianguliceps*, *D. marginatus*) of the Central German Uplands and Luxembourg, with *I. hexagonus* adults and nymphs dominating the findings, followed by adult *I. canisuga* and *I*. *ricinus* nymphs. This was particularly true for sites known as shelters or overwintering places of their preferred hosts, e.g., carnivorous mammals (red foxes and badgers) or hedgehogs (Arthur 1953; Harris and Thompson 1978). However, at this point, it also must be noted that a few (or even single) sampling events or underground sites can bias the proportions of individual tick species in the overall dataset. For example, 46% of all *I. canisuga* specimens collected in Germany originated from the 7 March 1998 collection event in the JOMI-Höhle, or in the case of *I. hexagonus* specimens from Luxembourg, 37% were collected in winter 2010/11 in the Méischtrefer Hiel. Only *I. ricinus* was found in tick samples from springs in the Central German Uplands. This species was occasionally encountered in underground sites, both in Germany (n = 77) and Luxembourg (7). The findings of *I. canisuga* and *I. trianguliceps* in Luxembourg constitute the first records for that country, thereby adding to the known tick fauna of Luxembourg.

Particularly noteworthy discoveries include a single nymph of the bat tick *I. ariadnae*, found on 01.02.2021 on the wall of a water passage in Friedewald (Hesse, Germany). The initial morphological identification was confirmed by sequencing its 16S rDNA marker. The specimen of our study showed 100% genetic sequence identity to *I. ariadnae* specimens collected in Germany, Hungary and Turkey (Hornok et al. 2015a, b; Hekimoglu et al. 2022). We recorded the bat species *Plecotus auritus*, *Myotis myotis* and *Myotis nattereri* at the same sampling site where *I. ariadnae* was found. *Ixodes ariadnae* is known to be associated with various bat species (Hornok et al. 2014; Sándor et al. 2019), including *P. auritus* and *Myotis* spp. This finding represents only the second report of this bat tick species in Germany. Given the relatively recent discovery and formal taxonomic description of *I. ariadnae* (Hornok et al. 2014), it can be expected that the species is probably more widely distributed in Germany than these two records indicate.

A single male of *D. marginatus* was found in a slate tunnel in Rhineland-Palatinate (Germany), on the right bank of the Rhine. Recent studies have shown that the distribution of *D. marginatus* in Central Europe has not changed much over the years and is relatively limited compared with *D. reticulatus* (Drehmann et al. 2020; Weigand et al. 2020; Rubel et al. 2021; Walter et al. 2016) determined that most of the Rhineland-Palatinate region is suitable habitat for *D. marginatus*, as this species prefers relatively dry and sparsely vegetated areas, often alongside rivers. A further westward range expansion also reaching Luxembourg can be expected in coming years.

Two rare findings of *I. trianguliceps* (2 females) were made in underground environments of Germany and Luxembourg. The mouse tick feeds exclusively on small animals (Brown et al. 2008) and is a very rare parasite of humans (Guglielmone and Robbins 2018). Most commonly, its life stages infest soricomorph mammals (Soricidae, Talpidae) and rodents (Cricetidae, Muridae), but this tick occasionally parasitizes other hosts, such as birds, sciurid rodents, chiropterans, and squamatans (Guglielmone et al. 2014; Kovalevskii et al. 2013). The species has a Palearctic distribution, occurring in many European countries and parts of northwestern Asia (Petney et al. 2012). However, georeferenced data for Central Europe are generally scarce (Estrada-Peña et al. 2018; Rubel et al. 2021).

### Ecological notes on the predominance of adult *Ixodes hexagonus* and *I. canisuga* in underground environments

Adults of *I. canisuga* and *I. hexagonus*, the two tick species most frequently represented by this stage in underground environments during our study, both mate off their hosts. Furthermore, *I. hexagonus* is known to detach from its hosts with the approach of darkness (Matuschka et al. 1990), and is quiescent for several days after molting. This behavior might explain why we encountered a much higher proportion of males than normally observed when directly collecting these two ticks from their preferred hosts (Arthur 1953; Harris and Thompson 1978; Walker 2018). Also, *I. hexagonus* was chiefly collected in non-natural subterranean environments in suburban areas of Luxembourg, which could be attributed to the ecology of its preferred host, hedgehogs, whose populations are often highest in suburban and urban areas because of reduced predation and higher food availability (Hubert et al. 2011; Walker 2018). This is also in accordance with the results of Neumann (1916), who recorded *I. vespertilionis* and *I. hexagonus* from 43 natural caves mostly located in France. However, only four specimens of *I. hexagonus* were recorded, suggesting that this species may indeed be more likely to occur in non-natural subterranean environments, which tend to be more common in (sub)urban areas and thus overlap with the environmental preferences of its preferred host. Red foxes (*Vulpes vulpes*) are the preferred final host for *I. canisuga*, but badgers (*Meles meles*), stone martens (*Martes foina*) or polecats (*Mustela putorius*) are also infested regularly. These hosts all have in common their use of self-constructed burrows or natural underground sites for shelter (e.g., crevices, holes, caves), which explains the high frequency of adult *I. canisuga* in the underground environments that we investigated. However, these data differ from those of Meyer-Kayser et al. (2012), who studied tick species and their developmental stages collected directly from red foxes in Thuringia, finding that adults of *I. ricinus* were most common (82% of all adult ticks), followed by nymphs of *I. canisuga* and *I. hexagonus* (85% of all nymphs). Notably, only a single male, but 485 female *I. canisuga* (ratio 0.2/99.8), and four males and 299 females of *I. hexagonus* (ratio 1.3/98.7) were detected among the 13,227 ticks collected from red foxes. Such results sharply contrast with our study of these two tick species when collected from underground environments, with gender ratios of 0.41/0.59 and 0.58/0.42 for *I. canisuga* and *I. hexagonus*, respectively. This indicates that collecting ticks from underground environments represents a promising strategy for obtaining males of these two species. Our results also suggest that *I. ricinus* adults apparently do not survive well in subterranean habitats, as they only comprised 7% of all adult ticks, but 47% of all nymphs and larvae.

### Co-occurrences of ticks in underground environments

The importance of spatio-temporal co-occurrences of tick species must be highlighted, such as in the case of the JOMI-Höhle in Rhineland-Palatinate (Germany), where *I. canisuga*, *I. hexagonus*, and *I. ricinus* were collected on a single day. In Luxembourg, all three species were also detected at single sites, but not on the same day, e.g., in the Fusselach (Mamerlayen) during summer 2007 and in the Méischtrefer Hiel during summer 2010. Co-occurrences of tick species can favor occasional transfers between hosts (Jahfari et al. 2017) and point to the importance of conducting further tick surveys in underground environments. This is relevant to public health, as *I. hexagonus* and *I. canisuga* may act as vectors of *Borrelia burgdorferi* and can transmit *Babesia missiroli* and tick-borne encephalitis (TBE) virus, respectively (Gern et al. 1991; Labuda and Randolph 1999; Skuballa et al. 2007; Jahfari et al. 2017; Arthur 1953) noted the risk of tick infestation at underground sites frequently visited by humans, such as air-raid shelters and similar constructions, especially if such sites are inhabited by *I. hexagonus*, which is a more frequent parasite of humans and pet animals than *I. canisuga* (Liebisch and Walter 1986; Guglielmone and Robbins 2018). The underground sites of the Mamerlayen in Luxembourg are known tourist attractions, so that the risk of tick bite there should be emphasized. Finally, as already stated by Jahfari et al. (2017), hedgehogs must be seen as a central element in the spread of ticks and tick-borne diseases, especially in urban and suburban settings.

### Sampling in underground environments

Tick populations are mostly monitored using dragging or flagging, methods that involve moving white fabric over vegetation to collect questing ticks. Alternatively, ticks can be collected from captured animals or from carcasses. Specimens may also be collected using dry ice traps (Yans et al. 2022). Flagging/dragging is highly effective when working with exophilic ticks, such as *I. ricinus*, but it is ineffective when seeking endophilic species, some of which are frequent in subterranean environments, as demonstrated by the results of our study. Host animals that visit these biotopes, either temporarily or for longer periods (e.g., for hibernation or dormancy), may leave detached endophilic species where they can be relatively easily collected, thereby adding to our knowledge of their distribution. A limitation to this approach is determining the hosts of the encountered specimens – without blood meal analysis. Exceptions to this are bat-infesting ticks, which are highly host specific (Sándor et al. 2019). Additionally, collecting in subterranean environments lacks a temporal dimension, as it is not possible to determine when tick specimens have dropped from their hosts after feeding. A tick found in a cave in early spring may have dropped from an active host just days before or months ago, while the host was hibernating (e.g., hedgehog or bats), in winter dormancy (e.g., badger) or resting (e.g., fox). Adding to this, larvae can be very long-lived, and prolonged periods of molting are observed under low temperatures, e.g., a mean of 60 days at 15 °C in a saturated atmosphere for *I. hexagonus* (Arthur 1951). Given that mean temperatures in Central European caves are between 8 and 10 °C (Zaenker et al. 2020), an even longer time period can be expected.

## Conclusions

Caves, mines, tunnels and other smaller underground cavities are often used as shelters or resting places (incl. hibernation and winter dormancy) by small to large mammals and their associated parasites and, as such, can contribute to the spread of ticks and tick-borne diseases. Despite limitations when collecting ticks in underground environments, our study results highlight the importance of obtaining taxonomic and distributional data from these often neglected habitats. Adult ticks of both sexes of *I. hexagonus* and *I. canisuga*, as well as nymphs of *I. ricinus*, were the most abundant specimens, but likewise rare findings were made (e.g., *I. ariadnae*, *I. trianguliceps*). The data at hand suggest that underground environments could act as reservoirs and sites of multiple tick-borne diseases by co-locating different tick species and their suitable hosts. Collecting ticks in such environments may help to increase our knowledge about the distribution of individual tick species, alongside classical flagging/dragging, thus gaining further insights into their ecologies and spatial dynamics – especially in suburban areas. Hedgehogs and their associated tick community might play a key role here.

## Electronic supplementary material

Below is the link to the electronic supplementary material.

Supplementary Material 1

Supplementary Material 2

Supplementary Material 3
