# Supplementary material for: Tick findings from subterranean environments in the Central German Uplands and Luxembourg reveal a predominance of male Ixodes hexagonus
Source: Exp Appl Acarol. 2023 Apr 28;89(3-4):461–73. doi: 10.1007/s10493-023-00795-2 (PMC10167134; doi:10.1007/s10493-023-00795-2)
Supplement: Supplementary file 1 — Supplementary Material 1 [file 10493_2023_795_MOESM1_ESM.docx]

**S1. Tick specimens from underground environments in Germany and Luxembourg.** Abbreviations: m = males, f = females, n = nymphs, l = larvae; hc = hand collection, bt = barber trap.

| **species** | **reference number** | **m** | **f** | **n** | **l** | **object name** | **country** | **date** | **method** |
| --- | --- | --- | --- | --- | --- | --- | --- | --- | --- |
| *Dermacentor marginatus* | Mi3530 | 1 |  |  |  | Schenkelbachstollen | Germany | 01/03/2012 | hc |
| *Ixodes ariadnae* | Mi5737 |  |  | 1 |  | Bachdurchlass Kothebachtal | Germany | 01/02/2021 | hc |
| *Ixodes canisuga* | Mi123 | 10 | 18 |  |  | JOMI-Höhle | Germany | 07/03/1998 | hc |
| *Ixodes canisuga* | MNHNL130195 |  |  | 1 |  | Stockfeldstollen 2 | Germany | 01/06/2003 | bt |
| *Ixodes canisuga* | Mi715 |  | 1 |  |  | Witzenhausener Höhle | Germany | 24/08/2003 | hc |
| *Ixodes canisuga* | Mi908 | 1 |  |  |  | Aachener Schanze Stollen 1 | Germany | 02/05/2004 | hc |
| *Ixodes canisuga* | MNHNL130074 | 1 |  |  |  | Oberst-Mark-Stollen | Germany | 03/07/2004 | hc |
| *Ixodes canisuga* | MNHNL130206 |  | 1 |  |  | Limbergstollen | Germany | 21/08/2004 | hc |
| *Ixodes canisuga* | MNHNL130214 | 1 |  |  |  | Limbergstollen | Germany | 30/04/2006 | bt |
| *Ixodes canisuga* | MNHNL130085 |  | 1 |  |  | Schlangenhöhle | Germany | 25/05/2006 | hc |
| *Ixodes canisuga* | MNHNL130104 |  | 1 |  |  | Schlangenhöhle | Germany | 25/05/2006 | hc |
| *Ixodes canisuga* | MNHNL130086 |  | 1 |  |  | Gipsgrube Eimersdorf | Germany | 04/06/2006 | hc |
| *Ixodes canisuga* | MNHNL130089 |  |  | 1 |  | Gipsgrube Eimersdorf | Germany | 08/07/2006 | bt |
| *Ixodes canisuga* | Mi1743 | 1 |  |  |  | Kurprinz-Friedrich-Wilhelm-Stollen | Germany | 11/11/2006 | hc |
| *Ixodes canisuga* | MNHNL130220 |  | 1 |  |  | Grande Fleur | Luxembourg | 20/05/2007 | hc |
| *Ixodes canisuga* | MNHNL130221 |  | 1 |  |  | Grande Fleur | Luxembourg | 20/05/2007 | hc |
| *Ixodes canisuga* | MNHNL130222 | 1 |  |  |  | Fusselach | Luxembourg | 27/05/2007 | hc |
| *Ixodes canisuga* | MNHNL130092 | 1 |  |  |  | Fusselach | Luxembourg | 17/08/2007 | hc |
| *Ixodes canisuga* | MNHNL130094 |  |  |  | 1 | Fusselach | Luxembourg | 30/12/2007 | bt |
| *Ixodes canisuga* | MNHNL130229 |  | 1 |  |  | Bitzmaschinn | Luxembourg | 01/05/2008 | hc |
| *Ixodes canisuga* | MNHNL130241 | 1 |  |  |  | Grube Moselberg 40 | Germany | 01/02/2009 | hc |
| *Ixodes canisuga* | Mi2682 |  | 1 |  |  | Wurzelhöhle | Germany | 28/12/2009 | hc |
| *Ixodes canisuga* | MNHNL130248 |  | 1 |  |  | Grube Querenberg | Germany | 21/03/2010 | hc |
| *Ixodes canisuga* | MNHNL130141 |  | 1 |  |  | Méischtrefer Hiel | Luxembourg | 27/03/2010 | bt |
| *Ixodes canisuga* | MNHNL130251 | 1 |  |  |  | Karsthöhle | Germany | 05/04/2010 | hc |
| *Ixodes canisuga* | MNHNL130150 |  | 1 |  |  | Méischtrefer Hiel | Luxembourg | 28/07/2010 | bt |
| *Ixodes canisuga* | MNHNL130152 |  |  |  | 5 | Morschstollen 1 | Germany | 28/08/2010 | bt |
| *Ixodes canisuga* | MNHNL130162 |  | 1 |  |  | Méischtrefer Hiel | Luxembourg | 06/11/2010 | bt |
| *Ixodes canisuga* | MNHNL130176 |  | 1 |  |  | Schlangenhöhle | Germany | 30/04/2011 | bt |
| *Ixodes canisuga* | MNHNL130177 |  | 1 |  |  | Schlangenhöhle | Germany | 30/04/2011 | hc |
| *Ixodes canisuga* | MNHNL130178 | 1 |  |  |  | Schlangenhöhle | Germany | 30/04/2011 | hc |
| *Ixodes canisuga* | MNHNL130179 | 1 |  |  |  | Schlangenhöhle | Germany | 30/04/2011 | hc |
| *Ixodes canisuga* | MNHNL130180 |  | 1 |  |  | Schlangenhöhle | Germany | 30/04/2011 | bt |
| *Ixodes canisuga* | MNHNL130181 |  | 1 |  |  | Schlangenhöhle | Germany | 30/04/2011 | bt |
| *Ixodes canisuga* | MNHNL130268 | 1 |  |  |  | Schlangenhöhle | Germany | 30/04/2011 | hc |
| *Ixodes canisuga* | MNHNL130184 |  | 1 |  |  | Stockfeldstollen 1 | Germany | 26/02/2012 | hc |
| *Ixodes canisuga* | MNHNL130187 | 1 |  |  |  | Luftschutzstollen Reisberg | Germany | 20/03/2012 | hc |
| *Ixodes canisuga* | MNHNL130272 | 1 |  |  |  | Luftschutzstollen Reisberg | Germany | 20/03/2012 | hc |
| *Ixodes canisuga* | Mi3565 | 1 |  | 1 |  | Große Rambacher Höhle | Germany | 28/12/2012 | hc |
| *Ixodes canisuga* | MNHNL130301 |  |  | 1 |  | Luftschutzstollen Reisberg | Germany | 05/01/2013 | hc |
| *Ixodes canisuga* | MNHNL130276 | 1 |  |  |  | Déiwepetz | Luxembourg | 09/03/2013 | hc |
| *Ixodes hexagonus* | MNHNL130108 |  | 1 |  |  | Petite Fleure | Luxembourg | 14/01/1996 | hc |
| *Ixodes hexagonus* | Mi111 | 1 |  |  |  | Meißelhöhle | Germany | 27/08/1997 | hc |
| *Ixodes hexagonus* | Mi123 | 2 | 2 | 2 |  | JOMI-Höhle | Germany | 07/03/1998 | hc |
| *Ixodes hexagonus* | MNHNL130109 | 1 |  |  |  | Windbergstollen | Germany | 04/02/1999 | hc |
| *Ixodes hexagonus* | Mi290 |  | 1 |  |  | Untere Nashornspalte | Germany | 31/07/2001 | hc |
| *Ixodes hexagonus* | MNHNL130069 | 1 |  |  |  | Büschfeld Versuchsstollen | Germany | 08/06/2003 | hc |
| *Ixodes hexagonus* | MNHNL130116 |  |  | 1 |  | Grube 1 im Potsch-Berg | Germany | 05/07/2003 | bt |
| *Ixodes hexagonus* | MNHNL130110 | 2 |  |  |  | Fritz-Grashoff-Stollen | Germany | 11/10/2003 | hc |
| *Ixodes hexagonus* | MNHNL130071 |  | 1 |  |  | Büschfeld Versuchsstollen | Germany | 01/11/2003 | hc |
| *Ixodes hexagonus* | MNHNL130202 |  |  |  | 1 | Büschfeld Überlosheimer Seite | Germany | 01/11/2003 | bt |
| *Ixodes hexagonus* | MNHNL130203 | 2 | 1 |  |  | Bei der Leienkaul 2 | Germany | 28/12/2003 | hc |
| *Ixodes hexagonus* | MNHNL130204 |  | 1 |  |  | Stollen W 652.2 | Germany | 25/02/2004 | hc |
| *Ixodes hexagonus* | Mi806 |  | 1 |  |  | Zyklon-Schacht | Germany | 06/03/2004 | hc |
| *Ixodes hexagonus* | MNHNL130117 | 1 |  |  |  | Stollen beim Teufelsfelsen | Germany | 03/04/2004 | hc |
| *Ixodes hexagonus* | MNHNL130075 | 1 |  |  |  | Friedrichsfeld 7 | Germany | 18/10/2004 | hc |
| *Ixodes hexagonus* | MNHNL130118 | 1 |  |  |  | Kalkgrube 1 Wackenmühle | Germany | 31/10/2004 | hc |
| *Ixodes hexagonus* | MNHNL130101 | 1 |  |  |  | Beckinger Buchwaldstollen | Germany | 01/11/2004 | hc |
| *Ixodes hexagonus* | Mi1119 |  |  | 1 |  | Grube Gottesgabe S-Lager, Hauptstollen | Germany | 13/03/2005 | hc |
| *Ixodes hexagonus* | Mi1143 | 1 |  |  |  | Witzenhausener Höhle | Germany | 19/05/2005 | hc |
| *Ixodes hexagonus* | MNHNL130080 |  |  | 1 |  | Auf dem Knopf | Germany | 06/06/2005 | hc |
| *Ixodes hexagonus* | MNHNL130083 | 1 |  |  |  | Auf dem Knopf | Germany | 17/09/2005 | hc |
| *Ixodes hexagonus* | MNHNL130103 | 1 |  |  |  | Auf dem Knopf | Germany | 17/09/2005 | hc |
| *Ixodes hexagonus* | MNHNL130211 |  | 1 |  |  | Stollen 144 | Germany | 17/09/2005 | bt |
| *Ixodes hexagonus* | MNHNL130076 |  | 1 |  |  | Rondel 1 | Germany | 22/10/2005 | hc |
| *Ixodes hexagonus* | MNHNL130077 | 1 |  |  |  | Guter Gang III mittlerer Stollen | Germany | 22/10/2005 | hc |
| *Ixodes hexagonus* | MNHNL130111 | 2 |  |  |  | Limbergstollen | Germany | 22/02/2006 | hc |
| *Ixodes hexagonus* | Mi1756 |  | 1 |  |  | Gertrudenstollen | Germany | 16/12/2006 | hc |
| *Ixodes hexagonus* | MNHNL130105 | 1 |  |  |  | Stollen Mörsbacher Strasse | Germany | 04/02/2007 | hc |
| *Ixodes hexagonus* | MNHNL130119 | 1 |  |  |  | Gratte Coude | Luxembourg | 17/02/2007 | hc |
| *Ixodes hexagonus* | MNHNL130120 | 1 | 1 |  |  | Gratte Coude | Luxembourg | 17/02/2007 | hc |
| *Ixodes hexagonus* | MNHNL130121 |  | 1 |  |  | Gratte Coude | Luxembourg | 17/02/2007 | hc |
| *Ixodes hexagonus* | MNHNL130122 |  | 1 |  |  | Stollen 1 im Buchenwald | Germany | 17/03/2007 | bt |
| *Ixodes hexagonus* | MNHNL130218 | 1 |  |  |  | Unbekannt 12 | Luxembourg | 07/04/2007 | hc |
| *Ixodes hexagonus* | MNHNL130124 |  |  | 3 |  | Minière Laangebierg Italien I | Luxembourg | 18/05/2007 | hc |
| *Ixodes hexagonus* | MNHNL130125 | 1 | 4 |  |  | Minière Laangebierg Italien I | Luxembourg | 18/05/2007 | hc |
| *Ixodes hexagonus* | MNHNL130223 | 1 | 1 | 1 | 1 | Fusselach | Luxembourg | 27/05/2007 | hc |
| *Ixodes hexagonus* | MNHNL130106 |  |  | 1 |  | Ahringstollen 4 | Germany | 02/06/2007 | bt |
| *Ixodes hexagonus* | MNHNL130126 | 1 |  |  |  | Ahringstollen 4 | Germany | 02/06/2007 | bt |
| *Ixodes hexagonus* | MNHNL130127 |  | 1 |  |  | Minière Laangebierg Italien I | Luxembourg | 15/08/2007 | bt |
| *Ixodes hexagonus* | MNHNL130128 | 2 | 3 | 1 |  | Minière Laangebierg Italien I | Luxembourg | 15/08/2007 | hc |
| *Ixodes hexagonus* | MNHNL130225 | 1 |  |  |  | Minière Laangebierg Italien I | Luxembourg | 15/08/2007 | bt |
| *Ixodes hexagonus* | MNHNL130227 |  |  |  | 1 | Fusselach | Luxembourg | 07/10/2007 | bt |
| *Ixodes hexagonus* | MNHNL130228 | 1 |  | 1 |  | Fusselach | Luxembourg | 07/10/2007 | hc |
| *Ixodes hexagonus* | MNHNL130129 |  |  | 1 |  | Minière Laangebierg Italien I | Luxembourg | 09/10/2007 | bt |
| *Ixodes hexagonus* | MNHNL130130 | 1 |  |  |  | Minière Laangebierg Italien I | Luxembourg | 09/10/2007 | hc |
| *Ixodes hexagonus* | MNHNL130113 | 1 |  |  |  | Minière Laangebierg Italien I | Luxembourg | 13/03/2008 | hc |
| *Ixodes hexagonus* | MNHNL130230 |  |  | 1 |  | Bitzmaschinn | Luxembourg | 01/05/2008 | bt |
| *Ixodes hexagonus* | MNHNL130231 |  | 1 |  |  | Fusselach | Luxembourg | 02/05/2008 | hc |
| *Ixodes hexagonus* | MNHNL130131 | 1 |  |  |  | Minière Laangebierg Italien I | Luxembourg | 10/05/2008 | hc |
| *Ixodes hexagonus* | MNHNL130232 |  | 1 |  |  | Minière Laange Gronn IV | Luxembourg | 11/05/2008 | hc |
| *Ixodes hexagonus* | MNHNL130132 |  | 1 |  |  | F 537 Birkenbacherhof | Germany | 01/06/2008 | hc |
| *Ixodes hexagonus* | MNHNL130233 | 1 |  |  |  | Karsthöhle | Germany | 07/06/2008 | hc |
| *Ixodes hexagonus* | MNHNL130133 | 1 |  |  |  | Minière Laangebierg Italien I | Luxembourg | 29/07/2008 | bt |
| *Ixodes hexagonus* | MNHNL130235 |  | 1 |  |  | Minière Laange Gronn IV | Luxembourg | 29/07/2008 | bt |
| *Ixodes hexagonus* | MNHNL130236 | 1 |  |  |  | Minière Laange Gronn IV | Luxembourg | 29/07/2008 | bt |
| *Ixodes hexagonus* | MNHNL130237 |  | 1 |  |  | Minière Laange Gronn IV | Luxembourg | 29/07/2008 | hc |
| *Ixodes hexagonus* | MNHNL130300 | 1 |  |  |  | Minière Laange Gronn IV | Luxembourg | 29/07/2008 | bt |
| *Ixodes hexagonus* | Mi2266 | 1 |  |  |  | Kielforsthöhle 3 | Germany | 22/09/2008 | hc |
| *Ixodes hexagonus* | MNHNL130096 | 1 |  | 1 |  | Minière Laange Gronn IV | Luxembourg | 28/12/2008 | bt |
| *Ixodes hexagonus* | MNHNL130240 | 1 |  |  |  | Minière Laange Gronn IV | Luxembourg | 28/12/2008 | bt |
| *Ixodes hexagonus* | MNHNL130242 |  |  | 1 |  | Gudrun-Diehl-Keller | Germany | 29/03/2009 | bt |
| *Ixodes hexagonus* | MNHNL130135 |  |  | 1 |  | Minière Laangebierg Italien II | Luxembourg | 19/08/2009 | bt |
| *Ixodes hexagonus* | MNHNL130245 | 1 |  |  |  | Minière Laangebierg Italien II | Luxembourg | 19/08/2009 | bt |
| *Ixodes hexagonus* | MNHNL130136 | 1 |  |  |  | Kelsbaach | Luxembourg | 20/08/2009 | bt |
| *Ixodes hexagonus* | MNHNL130247 |  |  | 1 |  | Gipsminn bei Girsterklaus | Luxembourg | 01/11/2009 | bt |
| *Ixodes hexagonus* | MNHNL130107 | 1 |  |  |  | Fort Louvigny | Luxembourg | 14/11/2009 | bt |
| *Ixodes hexagonus* | MNHNL130138 |  | 1 |  |  | Méischtrefer Hiel | Luxembourg | 31/12/2009 | hc |
| *Ixodes hexagonus* | MNHNL130139 | 3 |  | 1 |  | Méischtrefer Hiel | Luxembourg | 27/03/2010 | bt |
| *Ixodes hexagonus* | MNHNL130140 |  | 1 | 1 |  | Méischtrefer Hiel | Luxembourg | 27/03/2010 | bt |
| *Ixodes hexagonus* | MNHNL130142 |  | 2 |  |  | Méischtrefer Hiel | Luxembourg | 27/03/2010 | bt |
| *Ixodes hexagonus* | MNHNL130143 | 1 |  |  |  | Méischtrefer Hiel | Luxembourg | 27/03/2010 | bt |
| *Ixodes hexagonus* | MNHNL130144 | 1 |  | 3 |  | Méischtrefer Hiel | Luxembourg | 27/03/2010 | bt |
| *Ixodes hexagonus* | MNHNL130249 | 2 |  |  |  | Méischtrefer Hiel | Luxembourg | 27/03/2010 | hc |
| *Ixodes hexagonus* | MNHNL130250 |  |  | 1 |  | Méischtrefer Hiel | Luxembourg | 27/03/2010 | bt |
| *Ixodes hexagonus* | MNHNL130145 |  | 1 |  |  | Gipsminn Bettendorf | Luxembourg | 10/04/2010 | hc |
| *Ixodes hexagonus* | MNHNL130252 | 1 |  |  |  | Gipsminn Bettendorf | Luxembourg | 10/04/2010 | hc |
| *Ixodes hexagonus* | MNHNL130253 | 1 |  |  |  | Neue Kluft | Germany | 18/04/2010 | hc |
| *Ixodes hexagonus* | MNHNL130146 | 6 | 1 | 2 | 2 | Fort Louvigny | Luxembourg | 26/07/2010 | bt |
| *Ixodes hexagonus* | MNHNL130147 |  | 1 | 1 |  | Méischtrefer Hiel | Luxembourg | 28/07/2010 | bt |
| *Ixodes hexagonus* | MNHNL130148 |  |  | 1 |  | Méischtrefer Hiel | Luxembourg | 28/07/2010 | bt |
| *Ixodes hexagonus* | MNHNL130149 |  |  | 1 |  | Méischtrefer Hiel | Luxembourg | 28/07/2010 | bt |
| *Ixodes hexagonus* | MNHNL130255 |  | 1 |  |  | Gipsminn Bettendorf | Luxembourg | 28/07/2010 | hc |
| *Ixodes hexagonus* | MNHNL130151 |  | 1 |  |  | Bierkeller im Galgenberg | Germany | 08/08/2010 | hc |
| *Ixodes hexagonus* | MNHNL130257 |  |  | 1 |  | Festungswerk 6472-2 | Germany | 08/08/2010 | bt |
| *Ixodes hexagonus* | MNHNL130258 |  | 1 |  |  | Karsthöhle | Germany | 19/08/2010 | bt |
| *Ixodes hexagonus* | MNHNL130259 |  | 1 |  |  | Karsthöhle | Germany | 19/08/2010 | bt |
| *Ixodes hexagonus* | MNHNL130261 | 1 |  |  |  | Grube Rohrbach 3 | Germany | 21/08/2010 | hc |
| *Ixodes hexagonus* | MNHNL130153 |  |  | 1 |  | Méischtrefer Hiel | Luxembourg | 06/11/2010 | bt |
| *Ixodes hexagonus* | MNHNL130154 | 1 |  | 1 |  | Méischtrefer Hiel | Luxembourg | 06/11/2010 | bt |
| *Ixodes hexagonus* | MNHNL130155 | 1 |  |  |  | Méischtrefer Hiel | Luxembourg | 06/11/2010 | bt |
| *Ixodes hexagonus* | MNHNL130156 |  | 2 |  |  | Méischtrefer Hiel | Luxembourg | 06/11/2010 | bt |
| *Ixodes hexagonus* | MNHNL130157 |  |  | 1 |  | Méischtrefer Hiel | Luxembourg | 06/11/2010 | bt |
| *Ixodes hexagonus* | MNHNL130158 |  | 1 | 1 |  | Méischtrefer Hiel | Luxembourg | 06/11/2010 | bt |
| *Ixodes hexagonus* | MNHNL130159 | 1 |  | 2 |  | Méischtrefer Hiel | Luxembourg | 06/11/2010 | bt |
| *Ixodes hexagonus* | MNHNL130160 | 2 | 3 | 1 |  | Méischtrefer Hiel | Luxembourg | 06/11/2010 | bt |
| *Ixodes hexagonus* | MNHNL130161 | 1 |  | 1 |  | Méischtrefer Hiel | Luxembourg | 06/11/2010 | bt |
| *Ixodes hexagonus* | MNHNL130163 | 1 |  |  |  | Méischtrefer Hiel | Luxembourg | 06/11/2010 | hc |
| *Ixodes hexagonus* | MNHNL130264 |  |  | 1 |  | Méischtrefer Hiel | Luxembourg | 06/11/2010 | bt |
| *Ixodes hexagonus* | MNHNL130164 | 1 |  | 3 |  | Fort Louvigny | Luxembourg | 08/11/2010 | bt |
| *Ixodes hexagonus* | MNHNL130165 | 1 |  |  |  | Fort Lambert | Luxembourg | 08/11/2010 | bt |
| *Ixodes hexagonus* | MNHNL130265 | 1 |  |  |  | Schlangenhöhle | Germany | 29/12/2010 | hc |
| *Ixodes hexagonus* | Mi3053 | 1 |  | 3 |  | Bierkeller bei Reckrod | Germany | 11/01/2011 | hc |
| *Ixodes hexagonus* | MNHNL130167 | 1 |  |  |  | Gipsminn Bettendorf | Luxembourg | 22/01/2011 | bt |
| *Ixodes hexagonus* | MNHNL130168 |  |  |  | 2 | Gipsminn Bettendorf | Luxembourg | 22/01/2011 | bt |
| *Ixodes hexagonus* | MNHNL130169 | 1 |  |  |  | Gipsminn Bettendorf | Luxembourg | 22/01/2011 | hc |
| *Ixodes hexagonus* | MNHNL130170 |  | 1 |  |  | Méischtrefer Hiel | Luxembourg | 22/01/2011 | bt |
| *Ixodes hexagonus* | MNHNL130171 | 2 |  |  |  | Méischtrefer Hiel | Luxembourg | 22/01/2011 | bt |
| *Ixodes hexagonus* | MNHNL130173 |  | 1 | 1 |  | Méischtrefer Hiel | Luxembourg | 22/01/2011 | bt |
| *Ixodes hexagonus* | MNHNL130266 |  |  |  | 3 | Méischtrefer Hiel | Luxembourg | 22/01/2011 | bt |
| *Ixodes hexagonus* | MNHNL130166 |  | 1 |  |  | Gipsminn Bettendorf | Luxembourg | 22/02/2011 | bt |
| *Ixodes hexagonus* | MNHNL130172 |  | 1 | 1 |  | Méischtrefer Hiel | Luxembourg | 22/02/2011 | bt |
| *Ixodes hexagonus* | MNHNL130174 | 1 |  |  |  | Méischtrefer Hiel | Luxembourg | 22/02/2011 | bt |
| *Ixodes hexagonus* | MNHNL130175 |  | 2 | 1 |  | Méischtrefer Hiel | Luxembourg | 22/02/2011 | bt |
| *Ixodes hexagonus* | MNHNL130267 | 1 |  |  |  | Wichtelcheslee | Luxembourg | 12/04/2011 | hc |
| *Ixodes hexagonus* | Mi3270 |  | 1 |  |  | Schlucht (Naturhöhle) | Germany | 13/11/2011 | hc |
| *Ixodes hexagonus* | MNHNL130269 | 1 |  |  |  | Stollen F 539a | Germany | 18/11/2011 | hc |
| *Ixodes hexagonus* | MNHNL130182 | 1 |  |  |  | Alschbacher Bierkeller | Germany | 23/12/2011 | hc |
| *Ixodes hexagonus* | MNHNL130183 | 1 |  |  |  | Stockfeldstollen 1 | Germany | 26/02/2012 | hc |
| *Ixodes hexagonus* | MNHNL130185 |  | 1 |  |  | Stockfeldstollen 1 | Germany | 26/02/2012 | hc |
| *Ixodes hexagonus* | MNHNL130186 | 1 |  |  |  | Stockfeldstollen 1 | Germany | 26/02/2012 | hc |
| *Ixodes hexagonus* | MNHNL130270 | 1 |  |  |  | Stockfeldstollen 1 | Germany | 26/02/2012 | hc |
| *Ixodes hexagonus* | MNHNL130271 |  | 1 |  |  | Luftschutzstollen Reisberg | Germany | 20/03/2012 | hc |
| *Ixodes hexagonus* | MNHNL130273 |  | 1 |  |  | Luftschutzstollen Reisberg | Germany | 20/03/2012 | hc |
| *Ixodes hexagonus* | MNHNL130188 |  | 2 |  |  | Ursulas Keller | Germany | 05/01/2013 | bt |
| *Ixodes hexagonus* | MNHNL130189 |  |  | 1 |  | Ursulas Keller | Germany | 05/01/2013 | bt |
| *Ixodes hexagonus* | MNHNL130190 |  |  | 1 |  | Ursulas Keller | Germany | 05/01/2013 | bt |
| *Ixodes hexagonus* | MNHNL130274 | 2 | 2 | 1 |  | Ursulas Keller | Germany | 05/01/2013 | bt |
| *Ixodes hexagonus* | MNHNL130275 | 1 |  |  |  | Luftschutzstollen Reisberg | Germany | 05/01/2013 | bt |
| *Ixodes hexagonus* | MNHNL130302 |  | 1 |  |  | Ursulas Keller | Germany | 05/01/2013 | hc |
| *Ixodes hexagonus* | MNHNL130303 |  | 1 |  |  | Ursulas Keller | Germany | 05/01/2013 | hc |
| *Ixodes hexagonus* | MNHNL130304 |  | 1 |  |  | Adolf-Hitler-Stollen | Germany | 12/01/2013 | hc |
| *Ixodes hexagonus* | MNHNL130277 | 1 |  |  |  | Verbindungsgang Rhamplateau-Grundschleuse | Luxembourg | 10/05/2013 | hc |
| *Ixodes hexagonus* | MNHNL130278 | 1 |  |  |  | Verbindungsgang Rhamplateau-Grundschleuse | Luxembourg | 10/05/2013 | hc |
| *Ixodes hexagonus* | MNHNL130305 |  | 1 |  |  | Oberes Eisenerzbergwerk | Germany | 08/06/2013 | hc |
| *Ixodes hexagonus* | MNHNL130279 | 1 |  |  |  | Galerie de Bockholtzermillen | Luxembourg | 15/08/2013 | hc |
| *Ixodes hexagonus* | MNHNL130280 |  | 1 |  |  | Galerie de Bockholtzermillen | Luxembourg | 15/08/2013 | hc |
| *Ixodes hexagonus* | MNHNL130282 | 1 |  |  |  | Déiwepetz | Luxembourg | 16/08/2013 | hc |
| *Ixodes hexagonus* | MNHNL130283 | 2 |  |  |  | Galerie de Bockholtzermillen | Luxembourg | 18/08/2013 | hc |
| *Ixodes hexagonus* | MNHNL130284 |  | 1 |  |  | Minière Reschelerkopp | Luxembourg | 10/11/2013 | hc |
| *Ixodes hexagonus* | MNHNL130306 |  | 1 |  |  | Festungswerk 9462c | Germany | 11/01/2014 | hc |
| *Ixodes hexagonus* | MNHNL130286 | 1 |  |  |  | Schiefergrouf vu Schläif II | Luxembourg | 27/03/2014 | hc |
| *Ixodes hexagonus* | MNHNL130287 |  | 1 |  |  | Verbindungsgang Rhamplateau-Grundschleuse | Luxembourg | 16/06/2014 | hc |
| *Ixodes hexagonus* | MNHNL130288 | 1 |  |  |  | Verbindungsgang Rhamplateau-Grundschleuse | Luxembourg | 16/06/2014 | hc |
| *Ixodes hexagonus* | MNHNL130289 |  | 1 |  |  | Verbindungsgang Rhamplateau-Grundschleuse | Luxembourg | 16/06/2014 | hc |
| *Ixodes hexagonus* | MNHNL130290 | 1 |  |  |  | Verbindungsgang Rhamplateau-Grundschleuse | Luxembourg | 16/06/2014 | hc |
| *Ixodes hexagonus* | MNHNL130291 | 1 |  |  |  | Verbindungsgang Rhamplateau-Grundschleuse | Luxembourg | 16/06/2014 | hc |
| *Ixodes hexagonus* | MNHNL130292 |  | 1 |  |  | Minière Saintignon | Luxembourg | 16/06/2014 | hc |
| *Ixodes hexagonus* | MNHNL130293 |  | 1 |  |  | Minière Saintignon | Luxembourg | 16/06/2014 | hc |
| *Ixodes hexagonus* | MNHNL130294 | 1 |  |  |  | Minière Saintignon | Luxembourg | 16/06/2014 | hc |
| *Ixodes hexagonus* | MNHNL130295 |  | 1 |  |  | Minière Saintignon | Luxembourg | 16/06/2014 | hc |
| *Ixodes hexagonus* | MNHNL130296 | 1 |  |  |  | Verbindungsgang Rhamplateau-Grundschleuse | Luxembourg | 16/06/2014 | hc |
| *Ixodes hexagonus* | MNHNL130297 |  | 1 |  |  | Verbindungsgang Rhamplateau-Grundschleuse | Luxembourg | 16/06/2014 | hc |
| *Ixodes hexagonus* | MNHNL67125 | 1 |  |  |  | Verbindungsgang Rhamplateau-Grundschleuse | Luxembourg | 16/06/2014 | bt |
| *Ixodes hexagonus* | MNHNL130298 | 1 |  |  |  | Pulverkammer | Luxembourg | 17/06/2014 | hc |
| *Ixodes hexagonus* | MNHNL130299 | 1 |  |  |  | Minière Reschelerkopp | Luxembourg | 18/06/2014 | bt |
| *Ixodes hexagonus* | Mi4023 | 1 |  |  |  | Schallsinger Höhle | Germany | 01/11/2014 | hc |
| *Ixodes hexagonus* | MNHNL130307 |  | 1 |  |  | Kerlinger Stollen | Germany | 08/12/2015 | hc |
| *Ixodes hexagonus* | Mi4647 | 1 |  |  |  | Rockensüßer Höhle | Germany | 21/01/2017 | hc |
| *Ixodes hexagonus* | Mi5928 |  | 1 |  |  | Große Rambacher Höhle | Germany | 30/12/2021 | hc |
| *Ixodes ricinus* | Mi123 |  |  | 1 |  | JOMI-Höhle | Germany | 07/03/1998 | hc |
| *Ixodes ricinus* | Mi907 | 1 |  |  |  | Loch Nr. 3 | Germany | 01/05/2004 | hc |
| *Ixodes ricinus* | Mi1287 |  | 2 | 1 |  | Felsenkeller am Rauschenberg | Germany | 12/10/2005 | hc |
| *Ixodes ricinus* | Mi1456 |  |  | 1 |  | Stollen 2 im Mattesegraben | Germany | 06/05/2006 | hc |
| *Ixodes ricinus* | Mi1751 | 1 | 1 |  |  | Kurprinz-Friedrich-Wilhelm-Stollen | Germany | 16/12/2006 | hc |
| *Ixodes ricinus* | Mi1857 | 1 |  |  |  | Erdfallhöhle am Kies | Germany | 11/05/2007 | hc |
| *Ixodes ricinus* | Mi1878 | 1 |  |  |  | Müllbruchhöhle | Germany | 18/05/2007 | hc |
| *Ixodes ricinus* | Mi2090 | 1 |  |  |  | Daisbacher Stollen 1 | Germany | 08/03/2008 | hc |
| *Ixodes ricinus* | Mi2297 |  |  | 1 |  | Werkerkopfstollen 4 | Germany | 24/08/2008 | hc |
| *Ixodes ricinus* | Mi3791 |  | 1 |  |  | Rainloch | Germany | 13/07/2013 | hc |
| *Ixodes ricinus* | Mi3820 |  |  | 1 |  | Haugenlochhöhle | Germany | 15/09/2013 | hc |
| *Ixodes ricinus* | Mi4516 |  |  | 1 |  | Kleine Höhle im Beilstein | Germany | 05/09/2016 | hc |
| *Ixodes ricinus* | Mi5055 |  |  | 1 |  | Fuchshöhle | Germany | 23/02/2018 | hc |
| *Ixodes ricinus* | Mi5077 |  |  | 1 |  | Landefeldhöhle 2 | Germany | 28/04/2018 | hc |
| *Ixodes ricinus* | Mi5236 |  |  | 1 |  | Kellerwald Banfe Nord Fuß | Germany | 18/05/2018 | hc |
| *Ixodes ricinus* | Mi5238 |  |  | 1 |  | Kellerwald Banfe Nord Fuß | Germany | 24/07/2018 | hc |
| *Ixodes ricinus* | MNHNL130068 |  |  | 1 |  | Almersberg-Südfelsen 2 | Germany | 26/04/2003 | hc |
| *Ixodes ricinus* | MNHNL130191 |  |  | 1 |  | Petronell Grube B/C - Süd | Germany | 01/05/2003 | hc |
| *Ixodes ricinus* | MNHNL130192 |  |  | 1 |  | Building 7242-512 am Entenstein | Germany | 11/05/2003 | hc |
| *Ixodes ricinus* | MNHNL130193 |  |  | 1 |  | Nathenkeller | Germany | 30/05/2003 | hc |
| *Ixodes ricinus* | MNHNL130194 |  |  | 1 |  | Felsdach im Blauwald | Germany | 31/05/2003 | hc |
| *Ixodes ricinus* | MNHNL130196 |  |  | 1 |  | Höhle 6 am Rothenborn | Germany | 14/06/2003 | hc |
| *Ixodes ricinus* | MNHNL130197 |  |  | 1 |  | Waschbachstollen 7 | Germany | 21/06/2003 | hc |
| *Ixodes ricinus* | MNHNL130198 |  |  | 1 |  | Kirschbaumstollen | Germany | 13/07/2003 | hc |
| *Ixodes ricinus* | MNHNL130070 |  |  | 1 |  | Kirschbaumstollen | Germany | 11/10/2003 | hc |
| *Ixodes ricinus* | MNHNL130098 |  |  | 1 |  | Stollen beim E-Werk | Germany | 30/10/2003 | bt |
| *Ixodes ricinus* | MNHNL130199 |  |  |  | 2 | Hotelstollen | Germany | 30/10/2003 | bt |
| *Ixodes ricinus* | MNHNL130200 |  |  |  | 2 | Hotelstollen | Germany | 30/10/2003 | bt |
| *Ixodes ricinus* | MNHNL130201 |  |  | 1 |  | Stollen beim E-Werk | Germany | 30/10/2003 | bt |
| *Ixodes ricinus* | MNHNL130099 | 1 |  |  |  | Felsstrassenstollen | Germany | 31/10/2003 | hc |
| *Ixodes ricinus* | MNHNL130100 |  |  |  | 1 | Eiferstollen 21 | Germany | 23/11/2003 | bt |
| *Ixodes ricinus* | MNHNL130072 | 1 |  |  |  | Oberer Stollen am Krummen Ellenb. | Germany | 01/05/2004 | hc |
| *Ixodes ricinus* | MNHNL130205 |  |  | 1 |  | Langentalstollen 3 | Germany | 20/05/2004 | bt |
| *Ixodes ricinus* | MNHNL130073 |  |  | 1 |  | Schäferfelsen | Germany | 23/05/2004 | hc |
| *Ixodes ricinus* | MNHNL130207 | 1 |  |  |  | Einsiedeleinkeller | Germany | 22/08/2004 | hc |
| *Ixodes ricinus* | MNHNL130208 |  |  |  | 1 | Auener Stollen 31 | Germany | 04/09/2004 | hc |
| *Ixodes ricinus* | MNHNL130209 |  |  | 1 |  | Fichtenbruch 1 | Germany | 04/09/2004 | hc |
| *Ixodes ricinus* | MNHNL130078 |  |  | 1 |  | Bambeth-Stollen | Germany | 25/10/2004 | bt |
| *Ixodes ricinus* | MNHNL130079 |  |  | 1 |  | Retmerstollen | Germany | 25/10/2004 | bt |
| *Ixodes ricinus* | MNHNL130102 | 1 |  |  |  | Kalkgrube 1 Wackenmühle | Germany | 28/12/2004 | bt |
| *Ixodes ricinus* | MNHNL130210 |  |  | 1 |  | Stollen 1 in der Rechts-Halde | Germany | 27/05/2005 | hc |
| *Ixodes ricinus* | MNHNL130081 |  |  | 1 | 2 | Mittlerer Grüner Löwe-Stollen | Germany | 26/06/2005 | bt |
| *Ixodes ricinus* | MNHNL130082 |  |  | 1 |  | Schieferstollen IV | Germany | 24/07/2005 | hc |
| *Ixodes ricinus* | MNHNL130212 |  |  |  | 1 | Stollen 1 in der Rechts-Halde | Germany | 17/09/2005 | bt |
| *Ixodes ricinus* | MNHNL130213 |  |  |  | 1 | Stollen 1 in der Rechts-Halde | Germany | 17/09/2005 | hc |
| *Ixodes ricinus* | MNHNL130084 |  | 1 |  |  | Kupferheckstollen | Germany | 01/10/2005 | bt |
| *Ixodes ricinus* | MNHNL130112 |  |  | 2 |  | Schlangenhöhle | Germany | 25/05/2006 | hc |
| *Ixodes ricinus* | MNHNL130215 |  |  |  | 1 | Schlangenhöhle | Germany | 25/05/2006 | bt |
| *Ixodes ricinus* | MNHNL130216 |  |  |  | 1 | Schlangenhöhle | Germany | 25/05/2006 | hc |
| *Ixodes ricinus* | MNHNL130114 |  |  | 1 |  | Stollen am Zollbahnhof | Germany | 01/07/2006 | bt |
| *Ixodes ricinus* | MNHNL130217 |  |  | 1 |  | Stollen am Krötenbruch | Germany | 01/07/2006 | hc |
| *Ixodes ricinus* | MNHNL130087 |  |  | 1 |  | Gipsgrube Eimersdorf | Germany | 08/07/2006 | bt |
| *Ixodes ricinus* | MNHNL130088 |  |  | 1 |  | Gipsgrube Eimersdorf | Germany | 08/07/2006 | bt |
| *Ixodes ricinus* | MNHNL130123 | 1 |  |  |  | Stollen beim Teufelsfelsen | Germany | 01/04/2007 | hc |
| *Ixodes ricinus* | MNHNL130219 |  | 1 |  |  | Gouffre Saint Paul | Luxembourg | 07/04/2007 | hc |
| *Ixodes ricinus* | MNHNL130090 |  |  | 1 |  | Schraussendurchgangshöhle | Germany | 12/05/2007 | hc |
| *Ixodes ricinus* | MNHNL130091 |  |  |  | 1 | Haspelfelsenhöhle | Germany | 30/06/2007 | bt |
| *Ixodes ricinus* | MNHNL130224 |  |  |  | 1 | Wasserfall im Branntweinfelsen | Germany | 30/06/2007 | bt |
| *Ixodes ricinus* | MNHNL130226 |  |  | 1 |  | Fusselach | Luxembourg | 17/08/2007 | bt |
| *Ixodes ricinus* | MNHNL130093 |  |  |  | 1 | Weisse Grube (Suchstollen) | Germany | 13/11/2007 | bt |
| *Ixodes ricinus* | MNHNL130234 |  |  | 1 |  | Quellhöhle | Germany | 08/06/2008 | hc |
| *Ixodes ricinus* | MNHNL130238 |  |  |  | 1 | Heidekopfstollen 1 | Germany | 14/09/2008 | bt |
| *Ixodes ricinus* | MNHNL130239 |  |  |  | 1 | Westwallstollen Bremmelsberg | Germany | 18/10/2008 | bt |
| *Ixodes ricinus* | MNHNL130134 |  |  |  | 1 | Minière Laange Gronn IV | Luxembourg | 28/12/2008 | bt |
| *Ixodes ricinus* | MNHNL130243 | 1 |  |  |  | Kelsbaach | Luxembourg | 04/04/2009 | hc |
| *Ixodes ricinus* | MNHNL130244 |  | 1 |  |  | Felsenkammer 3 | Germany | 16/05/2009 | hc |
| *Ixodes ricinus* | MNHNL130097 |  |  |  | 1 | Weiberbornstollen 2 | Germany | 03/10/2009 | bt |
| *Ixodes ricinus* | MNHNL130246 |  |  |  | 1 | Weiberbornstollen 2 | Germany | 03/10/2009 | bt |
| *Ixodes ricinus* | MNHNL130137 |  |  |  | 1 | Festungswerk 402 | Germany | 26/12/2009 | bt |
| *Ixodes ricinus* | MNHNL130254 |  |  | 1 |  | Kirchberger Bergstollen 3 | Germany | 22/05/2010 | hc |
| *Ixodes ricinus* | MNHNL130115 |  |  |  | 1 | Méischtrefer Hiel | Luxembourg | 28/07/2010 | bt |
| *Ixodes ricinus* | MNHNL130256 |  |  | 1 |  | Festungswerk 6472-1 | Germany | 08/08/2010 | hc |
| *Ixodes ricinus* | MNHNL130260 |  |  | 2 |  | Kirchberger Bergstollen 10 | Germany | 21/08/2010 | bt |
| *Ixodes ricinus* | MNHNL130262 |  |  |  | 1 | Gut Glück 7 | Germany | 28/08/2010 | bt |
| *Ixodes ricinus* | MNHNL130263 |  |  |  | 1 | Morschstollen 1 | Germany | 28/08/2010 | bt |
| *Ixodes ricinus* | MNHNL130281 |  |  |  | 1 | Fort Rubamprez | Luxembourg | 16/08/2013 | bt |
| *Ixodes ricinus* | MNHNL130285 | 1 |  |  |  | Minière Schifflange | Luxembourg | 05/03/2014 | bt |
| *Ixodes trianguliceps* | Mi678 |  | 1 |  |  | Grube Rabenzeche | Germany | 22/06/2003 | hc |
| *Ixodes trianguliceps* | MNHNL130095 |  | 1 |  |  | Minière Hutbierg | Luxembourg | 31/12/2007 | hc |
