# Supplementary material for: Tick findings from subterranean environments in the Central German Uplands and Luxembourg reveal a predominance of male Ixodes hexagonus
Source: Exp Appl Acarol. 2023 Apr 28;89(3-4):461–73. doi: 10.1007/s10493-023-00795-2 (PMC10167134; doi:10.1007/s10493-023-00795-2)
Supplement: Supplementary file 2 — Supplementary Material 2 [file 10493_2023_795_MOESM2_ESM.docx]

**Table S2. Overview of tick specimens analyzed for the 16S rDNA marker.**

| **reference number** | **comment** | **molecular ID** | **GenBank Acc. No.** |
| --- | --- | --- | --- |
| Mi123 | nymph | *Ixodes ricinus* | OQ615895 |
| Mi123m | male | *Ixodes canisuga* | OQ615891 |
| Mi123w | female | *Ixodes canisuga* | OQ615892 |
| MNHNL130285 | male, broken | *Ixodes ricinus* | OQ615902 |
| MNHNL130276 | male | *Ixodes canisuga* | OQ615888 |
| MNHNL130301 | nymph | *Ixodes canisuga* | OQ615889 |
| MNHNL130161 | nymph | *Ixodes hexagonus* | OQ615894 |
| MNHNL130089 | nymph | *Ixodes canisuga* | OQ615885 |
| MNHNL130152 | larva | *Ixodes canisuga* | OQ615886 |
| Mi715 | female | *Ixodes canisuga* | OQ615893 |
| Mi1934 | nymph | *Ixodes ricinus* | OQ615903 |
| Mi2682 | female | *Ixodes canisuga* | OQ615887 |
| Mi3053 | nymph | *Ixodes hexagonus* | OQ615895 |
| Mi3565 | nymph | *Ixodes canisuga* | OQ615890 |
| Mi4012 | female | *Ixodes ricinus* | OQ615904 |
| Mi4306 | nymph | *Ixodes ricinus* | OQ615898 |
| Mi5031 | male | *Ixodes ricinus* | OQ615900 |
| Mi5236 | nymph | *Ixodes ricinus* | OQ615901 |
| Mi5467 | nymph | *Ixodes ricinus* | OQ615899 |
| Mi5508 | nymph | *Ixodes ricinus* | OQ615897 |
| Mi5737 | nymph | *Ixodes ariadnae* | OQ615884 |
