# Supplementary material for: Tick findings from subterranean environments in the Central German Uplands and Luxembourg reveal a predominance of male Ixodes hexagonus
Source: Exp Appl Acarol. 2023 Apr 28;89(3-4):461–73. doi: 10.1007/s10493-023-00795-2 (PMC10167134; doi:10.1007/s10493-023-00795-2)
Supplement: Supplementary file 3 — Supplementary Material 3 [file 10493_2023_795_MOESM3_ESM.docx]

**Table S3. Hand-collected tick specimens from regular spring monitoring in the Rhön mountains (Germany).** Abbreviations: m = males, f = females, n = nymphs, l = larvae; hc = hand collection.

| **species** | **reference numbers** | **m** | **f** | **n** | **l** | **object name** | **date** |
| --- | --- | --- | --- | --- | --- | --- | --- |
| *Ixodes ricinus* | Mi117 |  | 1 |  |  | Fulda-Niesig, Königswarter Straße 2a | 03/07/1997 |
| *Ixodes ricinus* | Mi672 |  |  |  | 1 | Quelle am Elsebachstollen | 14/06/2003 |
| *Ixodes ricinus* | Mi897 |  | 2 |  |  | Salzquellgebiet Breitzbachmühle, Quellaustritt 05 | 04/05/2004 |
| *Ixodes ricinus* | Mi927 |  | 1 |  |  | Quelle 2 am Fischbach | 08/06/2004 |
| *Ixodes ricinus* | Mi984 | 1 |  |  |  | Quelle 3a | 13/08/2004 |
| *Ixodes ricinus* | Mi993 |  |  | 1 |  | Große Küche, Quelle 4 | 04/08/2004 |
| *Ixodes ricinus* | Mi1060 |  |  | 1 |  | Talgangquelle 1 | 21/10/2004 |
| *Ixodes ricinus* | Mi1097 | 1 |  |  |  | Glasebach-Talquelle | 06/04/2005 |
| *Ixodes ricinus* | Mi1099 |  |  | 1 |  | Elsebach-Talquelle 07 | 05/04/2005 |
| *Ixodes ricinus* | Mi1102 |  | 1 |  |  | Elsebach-Talquelle 10 | 05/04/2005 |
| *Ixodes ricinus* | Mi1125 | 1 | 4 | 1 |  | Grebenquelle 2 | 30/04/2005 |
| *Ixodes ricinus* | Mi1128 | 1 |  |  |  | Katharinenbach Quelle | 30/04/2005 |
| *Ixodes ricinus* | Mi1130 | 1 | 2 |  |  | Riedbachquelle | 22/04/2005 |
| *Ixodes ricinus* | Mi1131 | 2 |  |  |  | Quelle an der Schönen Aussicht | 22/04/2005 |
| *Ixodes ricinus* | Mi1164 |  | 2 |  |  | Schnellmichbach-Quelle 2 | 19/05/2005 |
| *Ixodes ricinus* | Mi1169 |  |  | 1 |  | Elsebachquelle | 15/06/2005 |
| *Ixodes ricinus* | Mi1170 |  |  | 1 |  | Brachtquelle 3 | 15/06/2005 |
| *Ixodes ricinus* | Mi1251 | 1 |  |  |  | Roßbergquelle 1 | 31/08/2005 |
| *Ixodes ricinus* | Mi1360 |  |  | 1 |  | Quelle 2 an der Hohen Hart | 18/10/2005 |
| *Ixodes ricinus* | Mi1451 |  |  | 1 |  | Glasebach-Talquelle | 03/05/2006 |
| *Ixodes ricinus* | Mi1459 |  |  | 4 |  | Quelle 2 beim Sauerbrunnen Ramschied | 06/05/2006 |
| *Ixodes ricinus* | Mi1466 |  | 1 |  |  | Spangenborn-Seitenquelle 1 | 06/05/2006 |
| *Ixodes ricinus* | Mi1494 |  |  | 1 |  | Quelle 4 im NSG Landecker Berg | 20/04/2006 |
| *Ixodes ricinus* | Mi1511 | 3 | 1 |  |  | Quelle 2 am Fischbach | 06/06/2006 |
| *Ixodes ricinus* | Mi1528 |  |  | 1 |  | Quelle 2 östlich der Barbarossaquelle | 20/04/2006 |
| *Ixodes ricinus* | Mi1559 |  | 1 |  |  | Kalksumpf Ausbach, Untere gefaßte Quelle 1 | 19/06/2006 |
| *Ixodes ricinus* | Mi1569 |  |  | 1 |  | Quelle im Roh | 19/06/2006 |
| *Ixodes ricinus* | Mi1591 |  | 1 | 1 |  | Winkelrainquelle 1 | 07/08/2006 |
| *Ixodes ricinus* | Mi1592 | 1 |  |  |  | Anzenwegquelle 22 | 05/06/2006 |
| *Ixodes ricinus* | Mi1601 |  | 1 |  |  | Anzenwegquelle 6 | 04/06/2006 |
| *Ixodes ricinus* | Mi1609 |  |  | 1 |  | Anzenwegquelle 14 | 05/06/2006 |
| *Ixodes ricinus* | Mi1612 |  | 1 |  |  | Mittelbergquelle | 13/07/2006 |
| *Ixodes ricinus* | Mi1625 |  |  | 1 |  | Quelle 4 am Großen Kopf | 27/07/2006 |
| *Ixodes ricinus* | Mi1628 |  |  | 1 |  | Geräumequelle 2 | 27/07/2006 |
| *Ixodes ricinus* | Mi1669 | 1 |  |  |  | Quelle 1 am Großen Buchberg | 05/05/2006 |
| *Ixodes ricinus* | Mi1679 |  |  | 1 |  | Querenberg-Quelle 5 | 30/09/2006 |
| *Ixodes ricinus* | Mi1844 |  |  | 1 |  | Karstquelle am Aubach, Quelle | 21/04/2007 |
| *Ixodes ricinus* | Mi1845 | 1 |  |  |  | Rendatalquelle IV | 01/04/2007 |
| *Ixodes ricinus* | Mi1864 |  |  | 1 |  | Kirschenborn | 11/05/2007 |
| *Ixodes ricinus* | Mi1868 | 1 |  |  |  | Querenbergquelle 50 | 13/05/2007 |
| *Ixodes ricinus* | Mi1873 |  | 2 |  |  | Dreibrunnen, Überlauf | 13/05/2007 |
| *Ixodes ricinus* | Mi1931 |  | 1 |  |  | Birkenrückquelle 1 | 01/01/2007 |
| *Ixodes ricinus* | Mi1934 |  | 1 | 1 |  | Bartenbachquelle 2 | 02/04/2007 |
| *Ixodes ricinus* | Mi1936 | 2 | 4 | 1 |  | Bartenbachquelle 3 | 02/04/2007 |
| *Ixodes ricinus* | Mi1945 |  |  | 1 |  | Bartenbachquelle 5 | 02/04/2007 |
| *Ixodes ricinus* | Mi1948 | 1 |  |  |  | Bartenbachquelle 17 | 02/04/2007 |
| *Ixodes ricinus* | Mi1994 |  |  | 1 |  | Eschelbachquelle 14 | 03/04/2007 |
| *Ixodes ricinus* | Mi2028 |  | 1 |  |  | Quelle 8 im Gebrannten | 09/10/2007 |
| *Ixodes ricinus* | Mi2031 |  |  | 1 |  | Hundsbachquelle 4 | 09/10/2007 |
| *Ixodes ricinus* | Mi2035 |  |  | 1 |  | Eschelbachquelle 7 | 03/04/2007 |
| *Ixodes ricinus* | Mi2036 |  |  | 1 |  | Eschelbachquelle 6 | 03/04/2007 |
| *Ixodes ricinus* | Mi2039 | 1 |  |  |  | Bartenbachquelle 9 | 02/04/2007 |
| *Ixodes ricinus* | Mi2041 |  |  | 2 |  | Bartenbachquelle 19 | 03/04/2007 |
| *Ixodes ricinus* | Mi2067 |  |  | 1 |  | Biebergrundquelle 2 | 15/10/2007 |
| *Ixodes ricinus* | Mi2102 |  | 1 |  |  | Raue-Hardt-Quelle | 15/03/2008 |
| *Ixodes ricinus* | Mi2129 | 1 |  |  |  | Quelle nördlich der Kummertsliede | 03/05/2008 |
| *Ixodes ricinus* | Mi2133 |  |  | 1 |  | Eubequelle 19 | 04/05/2008 |
| *Ixodes ricinus* | Mi2148 |  |  | 1 |  | Braunstein-Quelle | 31/03/2008 |
| *Ixodes ricinus* | Mi2284 |  |  | 1 |  | Frankfurt am Main, Griesheim, Elsterstr. 32 | 01/01/2008 |
| *Ixodes ricinus* | Mi2306 |  | 2 | 1 |  | Zeckenquelle | 10/10/2008 |
| *Ixodes ricinus* | Mi2308 |  |  | 1 |  | Ruhlauberquelle 15 | 09/10/2008 |
| *Ixodes ricinus* | Mi2314 |  |  | 1 |  | Quelle 7 am Heiligenstocktriesch | 09/10/2008 |
| *Ixodes ricinus* | Mi2319 |  |  | 1 |  | Geismarsbergquelle 3 | 09/10/2008 |
| *Ixodes ricinus* | Mi2338 |  |  | 1 |  | Hainchen-Quelle 6 | 10/10/2008 |
| *Ixodes ricinus* | Mi2446 |  |  | 1 |  | Kronbergquelle 3 | 17/04/2009 |
| *Ixodes ricinus* | Mi2468 |  |  | 1 |  | Wasserbergquelle 2 | 16/06/2008 |
| *Ixodes ricinus* | Mi2471 |  |  | 1 |  | Gerhardsbergquelle | 12/06/2008 |
| *Ixodes ricinus* | Mi2485 |  | 1 |  |  | Quelle beim Forsthaus Mellnau | 18/06/2008 |
| *Ixodes ricinus* | Mi2502 |  |  | 1 |  | Bloßenberg, Malaisefalle | 04/06/2007 |
| *Ixodes ricinus* | Mi2527 |  |  |  | 1 | Sickerquelle im Schloßpark des Schlosses Fasanerie | 05/08/2009 |
| *Ixodes ricinus* | Mi2665 |  |  | 1 |  | Quelle 27 im Krofdorfer Forst | 15/05/2008 |
| *Ixodes ricinus* | Mi2688 |  |  | 1 |  | Quelle über den Eisenlöchern | 27/08/2008 |
| *Ixodes ricinus* | Mi2694 | 1 |  |  |  | Keilquelle 4 | 26/08/2008 |
| *Ixodes ricinus* | Mi2695 |  |  | 1 |  | Keilquelle 4 | 26/08/2008 |
| *Ixodes ricinus* | Mi2722 |  | 1 |  |  | Steingrabenquelle 5 | 11/08/2008 |
| *Ixodes ricinus* | Mi2734 |  |  | 1 |  | Quelle 10 im Krofdorfer Forst | 04/03/2008 |
| *Ixodes ricinus* | Mi2735 |  |  | 1 |  | Quelle 17 im Krofdorfer Forst | 06/03/2008 |
| *Ixodes ricinus* | Mi2818 |  |  | 1 |  | Seitenquelle 5 am Zahlbach | 21/08/2008 |
| *Ixodes ricinus* | Mi2819 |  | 1 | 1 |  | Quelle 1 am Gebrannten Berg | 21/08/2008 |
| *Ixodes ricinus* | Mi2826 | 1 |  |  |  | Plätze-Nebenquelle 6 | 12/08/2009 |
| *Ixodes ricinus* | Mi2832 |  |  | 1 |  | Quelle 34 im Krofdorfer Forst | 11/04/2008 |
| *Ixodes ricinus* | Mi2837 |  |  | 1 |  | Quelle 2 im Krofdorfer Forst | 29/07/2008 |
| *Ixodes ricinus* | Mi2904 |  | 1 |  |  | Auersbergquelle 168 | 22/05/2010 |
| *Ixodes ricinus* | Mi2910 |  | 1 |  |  | Auersbergquelle 98 | 21/05/2010 |
| *Ixodes ricinus* | Mi3293 |  |  | 1 |  | Schlosspark des Schlosses Fasanerie, Bodenfallen | 16/06/2009 |
| *Ixodes ricinus* | Mi3546 |  |  | 1 |  | Kornbergquelle 1 | 25/07/2012 |
| *Ixodes ricinus* | Mi3551 |  |  |  | 1 | Kornbergquelle 20 | 18/09/2012 |
| *Ixodes ricinus* | Mi3728 |  | 1 |  |  | Schmerbachquelle 31 | 01/08/2013 |
| *Ixodes ricinus* | Mi3874 | 1 |  |  |  | Eifa-Nebenquelle 2 | 20/06/2014 |
| *Ixodes ricinus* | Mi3896 | 2 |  | 2 |  | Fahrentrieschquelle 2 | 10/05/2014 |
| *Ixodes ricinus* | Mi4012 |  | 1 |  |  | Fulda-Niesig, Königswarter Straße 2a | 26/04/2015 |
| *Ixodes ricinus* | Mi4013 | 1 |  |  |  | Fulda-Niesig, Königswarter Straße 2a | 30/04/2015 |
| *Ixodes ricinus* | Mi4066 | 1 |  |  |  | Arzwaldquelle 4 | 13/04/2015 |
| *Ixodes ricinus* | Mi4155 |  | 1 |  |  | Simmetquelle 2 | 10/04/2015 |
| *Ixodes ricinus* | Mi4157 | 1 | 1 |  |  | Simmetquelle 5 | 10/04/2015 |
| *Ixodes ricinus* | Mi4158 |  |  | 1 |  | Simmetquelle 6 | 10/04/2015 |
| *Ixodes ricinus* | Mi4257 | 1 |  |  |  | Quelle 2 an der Sandgrube | 03/08/2015 |
| *Ixodes ricinus* | Mi4291 |  | 1 |  |  | Hasenbachquelle 18 | 31/10/2014 |
| *Ixodes ricinus* | Mi4306 |  |  | 1 |  | Untere Steinboßquelle 5 | 21/09/2015 |
| *Ixodes ricinus* | Mi4307 |  |  | 1 |  | Schnabelsroth-Quelle 3 | 21/09/2015 |
| *Ixodes ricinus* | Mi4319 |  | 1 |  |  | Königsbergquelle 3 | 18/04/2015 |
| *Ixodes ricinus* | Mi4321 | 3 | 1 |  |  | Königsbergquelle 5 | 18/04/2015 |
| *Ixodes ricinus* | Mi4342 |  | 1 |  |  | Quelle 1 an der Ziegelhütte | 03/04/2016 |
| *Ixodes ricinus* | Mi4393 | 1 |  |  |  | Untere Horloffquelle | 22/05/2016 |
| *Ixodes ricinus* | Mi4412 |  |  | 1 |  | Quelle 3 im Buchenroder Graben | 01/07/2016 |
| *Ixodes ricinus* | Mi4472 |  | 1 |  |  | Quelle in der Aschstrut | 09/07/2016 |
| *Ixodes ricinus* | Mi4485 |  |  | 1 |  | Katharinenbach Quelle | 11/06/2016 |
| *Ixodes ricinus* | Mi4541 | 1 | 1 |  |  | Quelle 1 auf der Addau | 27/05/2016 |
| *Ixodes ricinus* | Mi4559 | 1 |  |  |  | Quelle nördlich des mittleren Forellenteichs | 28/05/2016 |
| *Ixodes ricinus* | Mi4602 |  |  |  | 1 | Walthersborn, obere Quellfassung | 29/05/2016 |
| *Ixodes ricinus* | Mi4603 |  |  | 1 |  | Quelle 1 am Sauborn | 29/05/2016 |
| *Ixodes ricinus* | Mi4605 |  | 1 |  |  | Hillersbach-Nebenquelle 1 | 29/05/2016 |
| *Ixodes ricinus* | Mi4607 |  |  | 1 |  | Hillersbach-Nebenquelle 3 | 29/05/2016 |
| *Ixodes ricinus* | Mi4618 |  |  | 1 |  | Höllerskopfquelle 1 | 26/05/2016 |
| *Ixodes ricinus* | Mi4714 | 1 |  |  |  | Altenkopfquelle 8 | 29/04/2017 |
| *Ixodes ricinus* | Mi4720 |  |  | 1 |  | Altenkopfquelle 1 | 29/04/2017 |
| *Ixodes ricinus* | Mi4721 |  | 1 |  |  | Quelle 9 südlich der Reifendorfer Mühle | 29/04/2017 |
| *Ixodes ricinus* | Mi4723 |  |  | 1 |  | Quelle 7 südlich der Reifendorfer Mühle | 29/04/2017 |
| *Ixodes ricinus* | Mi4817 |  | 1 |  |  | Maßborn 21 | 02/04/2017 |
| *Ixodes ricinus* | Mi4845 | 1 |  |  |  | Melpertser Hutequelle 37 | 30/04/2017 |
| *Ixodes ricinus* | Mi4866 |  | 1 |  |  | Thaidener Hutequelle 74 | 28/04/2017 |
| *Ixodes ricinus* | Mi4871 |  | 1 |  |  | Thaidener Hutequelle 54 | 28/04/2017 |
| *Ixodes ricinus* | Mi4883 |  | 1 |  |  | Thaidener Hutequelle 29 | 24/04/2017 |
| *Ixodes ricinus* | Mi4884 | 1 |  |  |  | Thaidener Hutequelle 30 | 24/04/2017 |
| *Ixodes ricinus* | Mi4901 |  |  | 1 |  | Thaidener Hutequelle 17 | 23/04/2017 |
| *Ixodes ricinus* | Mi4949 |  | 1 |  |  | Buchschirmquelle 76 | 13/04/2017 |
| *Ixodes ricinus* | Mi4950 |  | 1 |  |  | Buchschirmquelle 77 | 13/04/2017 |
| *Ixodes ricinus* | Mi4951 | 2 |  |  |  | Buchschirmquelle 78 | 13/04/2017 |
| *Ixodes ricinus* | Mi4960 | 1 |  |  |  | Buchschirmquelle 54 | 12/04/2017 |
| *Ixodes ricinus* | Mi4980 | 1 |  |  |  | Buchschirmquelle 14 | 11/04/2017 |
| *Ixodes ricinus* | Mi4982 |  | 1 |  |  | Buchschirmquelle 17 | 11/04/2017 |
| *Ixodes ricinus* | Mi4984 | 1 | 2 |  |  | Buchschirmquelle 22 | 11/04/2017 |
| *Ixodes ricinus* | Mi4987 | 1 |  |  |  | Buchschirmquelle 2 | 11/04/2017 |
| *Ixodes ricinus* | Mi4993 | 1 | 1 |  |  | Brückenhutquelle 35 | 09/04/2017 |
| *Ixodes ricinus* | Mi4996 | 1 |  |  |  | Brückenhutquelle 39 | 09/04/2017 |
| *Ixodes ricinus* | Mi5000 |  | 1 |  |  | Brückenhutquelle 46 | 09/04/2017 |
| *Ixodes ricinus* | Mi5008 | 1 |  |  |  | Brückenhutquelle 28 | 08/04/2017 |
| *Ixodes ricinus* | Mi5024 | 1 | 1 |  |  | Brückenhutquelle 13 | 08/04/2017 |
| *Ixodes ricinus* | Mi5027 |  | 1 |  |  | Brückenhutquelle 16 | 08/04/2017 |
| *Ixodes ricinus* | Mi5031 | 1 |  |  |  | Brückenhutquelle 20 | 08/04/2017 |
| *Ixodes ricinus* | Mi5032 | 1 |  |  |  | Brückenhutquelle 21 | 08/04/2017 |
| *Ixodes ricinus* | Mi5079 | 1 |  |  |  | Quelle 1 in der Grürmannsheide | 11/05/2018 |
| *Ixodes ricinus* | Mi5114 |  | 1 |  |  | Quelle 14 im Landkrankenhauswald | 03/04/2018 |
| *Ixodes ricinus* | Mi5197 | 2 | 1 |  |  | Banfetalquelle 3 | 07/04/2018 |
| *Ixodes ricinus* | Mi5205 | 1 |  | 1 |  | Kirchwegquelle 2 | 07/04/2018 |
| *Ixodes ricinus* | Mi5374 |  |  | 1 |  | Schlichtwasserquelle 14 | 22/03/2019 |
| *Ixodes ricinus* | Mi5409 | 1 | 1 |  |  | Untere Struttquelle 45 | 19/04/2019 |
| *Ixodes ricinus* | Mi5419 |  |  | 1 |  | Untere Struttquelle 25 | 18/04/2019 |
| *Ixodes ricinus* | Mi5425 | 1 |  |  |  | Untere Struttquelle 8 | 18/04/2019 |
| *Ixodes ricinus* | Mi5462 | 1 |  |  |  | Kirschenwegquelle 3 | 24/04/2019 |
| *Ixodes ricinus* | Mi5467 |  |  | 1 |  | Grützenbrunnen | 27/07/2019 |
| *Ixodes ricinus* | Mi5476 | 1 |  |  |  | Melpertser Hutequelle 47 | 06/04/2020 |
| *Ixodes ricinus* | Mi5487 | 1 | 2 | 1 |  | Fußlochquelle 35 | 24/04/2020 |
| *Ixodes ricinus* | Mi5496 |  | 1 |  |  | Fußlochquelle 22 | 23/04/2020 |
| *Ixodes ricinus* | Mi5506 |  |  | 1 |  | Fußlochquelle 4 | 23/04/2020 |
| *Ixodes ricinus* | Mi5507 | 1 |  |  |  | Fußlochquelle 3 | 23/04/2020 |
| *Ixodes ricinus* | Mi5508 |  |  | 1 |  | Fußlochquelle 1 | 23/04/2020 |
| *Ixodes ricinus* | Mi5530 | 1 | 2 | 1 |  | Weissenbrunnen 4 | 09/05/2020 |
| *Ixodes ricinus* | Mi5531 |  | 2 |  |  | Weissenbrunnen 6 | 09/05/2020 |
| *Ixodes ricinus* | Mi5538 | 1 |  |  |  | Bremer Hut-Quelle 3 | 20/04/2020 |
| *Ixodes ricinus* | Mi5543 | 1 |  |  |  | Grabentümpel 06 am Wadberg | 13/07/2020 |
| *Ixodes ricinus* | Mi5549 |  | 1 |  |  | Mannsbergquelle 16 | 21/04/2020 |
| *Ixodes ricinus* | Mi5554 |  | 2 |  |  | Bremer Hut-Quelle 37 | 21/04/2020 |
| *Ixodes ricinus* | Mi5556 | 1 | 1 |  |  | Bremer Hut-Quelle 1 | 20/04/2020 |
| *Ixodes ricinus* | Mi5561 |  |  | 1 |  | Quelle 2 am Bergwiesenwäldchen | 08/05/2020 |
| *Ixodes ricinus* | Mi5569 | 1 |  |  |  | Tannenhofquelle 14 | 08/05/2020 |
| *Ixodes ricinus* | Mi5577 |  | 1 |  |  | Bremer Hut-Quelle 18 | 20/04/2020 |
| *Ixodes ricinus* | Mi5593 |  |  | 1 |  | Bremer Hut-Quelle 22 | 20/04/2020 |
| *Ixodes ricinus* | Mi5595 | 2 |  |  |  | Bremer Hut-Quelle 20 | 20/04/2020 |
| *Ixodes ricinus* | Mi5597 |  | 1 |  |  | Bremer Hut-Quelle 10 | 20/04/2020 |
| *Ixodes ricinus* | Mi5601 |  | 1 |  |  | Bremer Hut-Quelle 19 | 20/04/2020 |
| *Ixodes ricinus* | Mi5607 | 1 |  |  |  | Aschach-Nebenquelle 8 | 09/05/2020 |
| *Ixodes ricinus* | Mi5610 |  |  | 1 |  | Aschach-Nebenquelle 12 | 09/05/2020 |
| *Ixodes ricinus* | Mi5625 |  |  | 1 |  | Aschach-Nebenquelle 13 | 09/05/2020 |
| *Ixodes ricinus* | Mi5632 |  |  | 1 |  | Aschach-Nebenquelle 11 | 09/05/2020 |
| *Ixodes ricinus* | Mi5640 | 1 |  |  |  | Tannenhofquelle 2 | 04/05/2020 |
| *Ixodes ricinus* | Mi5644 | 1 |  |  |  | Melpertser Hutequelle 85 | 09/04/2020 |
| *Ixodes ricinus* | Mi5647 | 1 |  |  |  | Melpertser Hutequelle 79 | 09/04/2020 |
| *Ixodes ricinus* | Mi5649 |  | 2 |  |  | Melpertser Hutequelle 82 | 09/04/2020 |
| *Ixodes ricinus* | Mi5650 | 1 |  |  |  | Melpertser Hutequelle 83 | 09/04/2020 |
| *Ixodes ricinus* | Mi5651 | 1 | 1 |  |  | Melpertser Hutequelle 61 | 07/04/2020 |
| *Ixodes ricinus* | Mi5655 |  | 1 |  |  | Buchenstrauchquelle 1 | 06/05/2020 |
| *Ixodes ricinus* | Mi5656 | 3 | 5 |  |  | Buchenstrauchquelle 3 | 06/05/2020 |
| *Ixodes ricinus* | Mi5660 |  | 1 |  |  | Tannenhofquelle 10 | 04/05/2020 |
| *Ixodes ricinus* | Mi5665 | 2 | 1 |  |  | Buchenstrauchquelle 4 | 06/05/2020 |
| *Ixodes ricinus* | Mi5666 |  | 1 |  |  | Buchenstrauchquelle 5 | 06/05/2020 |
| *Ixodes ricinus* | Mi5670 |  | 1 |  |  | Buchenstrauchquelle 10 | 06/05/2020 |
| *Ixodes ricinus* | Mi5672 |  |  | 1 |  | Buchenstrauchquelle 23 | 07/05/2020 |
| *Ixodes ricinus* | Mi5676 |  |  | 1 |  | Buchenstrauchquelle 29 | 07/05/2020 |
| *Ixodes ricinus* | Mi5679 | 1 |  |  |  | Melpertser Hutequelle 64 | 07/04/2020 |
| *Ixodes ricinus* | Mi5683 |  | 1 |  |  | Melpertser Hutequelle 55 | 07/04/2020 |
| *Ixodes ricinus* | Mi5684 |  | 1 |  |  | Melpertser Hutequelle 56 | 07/04/2020 |
| *Ixodes ricinus* | Mi5688 |  | 2 |  |  | Melpertser Hutequelle 62 | 07/04/2020 |
| *Ixodes ricinus* | Mi5690 | 1 |  |  |  | Melpertser Hutequelle 69 | 08/04/2020 |
| *Ixodes ricinus* | Mi5692 | 1 |  |  |  | Melpertser Hutequelle 71 | 08/04/2020 |
| *Ixodes ricinus* | Mi5770 |  | 1 |  |  | Lahrbachquelle | 07/04/2021 |
| *Ixodes ricinus* | Mi5774 |  |  | 1 |  | Fußlochquelle 76 | 25/04/2021 |
| *Ixodes ricinus* | Mi5775 |  |  | 1 |  | Fußlochquelle 79 | 25/04/2021 |
| *Ixodes ricinus* | Mi5778 | 1 |  |  |  | Fußlochquelle 89 | 25/04/2021 |
| *Ixodes ricinus* | Mi5780 |  |  | 1 |  | Fußlochquelle 80 | 25/04/2021 |
| *Ixodes ricinus* | Mi5783 |  | 1 |  |  | Fußlochquelle 49 | 24/04/2021 |
| *Ixodes ricinus* | Mi5788 | 1 |  |  |  | Fußlochquelle 82 | 25/04/2021 |
| *Ixodes ricinus* | Mi5793 | 1 |  |  |  | Fußlochquelle 51 | 24/04/2021 |
| *Ixodes ricinus* | Mi5799 |  |  | 2 |  | Staatsebachquelle 1 | 12/07/2021 |
| *Ixodes ricinus* | Mi5801 |  |  | 1 |  | Quelle 4 nördlich von Bieben | 24/07/2021 |
| *Ixodes ricinus* | Mi5804 | 1 |  |  |  | Fußlochquelle 61 | 24/04/2021 |
| *Ixodes ricinus* | Mi5805 |  |  | 1 |  | Fußlochquelle 62 | 24/04/2021 |
| *Ixodes ricinus* | Mi5809 | 3 |  | 1 |  | Fußlochquelle 66 | 24/04/2021 |
| *Ixodes ricinus* | Mi5810 |  | 1 | 5 |  | Fußlochquelle 67 | 24/04/2021 |
| *Ixodes ricinus* | Mi5811 | 1 |  |  |  | Fußlochquelle 68 | 24/04/2021 |
| *Ixodes ricinus* | Mi5814 | 1 |  | 1 |  | Fußlochquelle 86 | 25/04/2021 |
| *Ixodes ricinus* | Mi5869 |  |  | 1 |  | Quelle 13 südöstlich der Katzenhöhe | 09/05/2021 |
| *Ixodes ricinus* | Mi5880 |  |  | 1 |  | Kammbergquelle 5 | 11/06/2021 |
| *Ixodes ricinus* | Mi5887 |  | 1 |  |  | Tannenberg-Quelle 9 | 12/06/2021 |
| *Ixodes ricinus* | Mi5889 |  |  | 1 |  | Tannenberg-Quelle 16 | 12/06/2021 |
| *Ixodes ricinus* | Mi5896 |  | 1 |  |  | Kammbergquelle 20 | 11/06/2021 |
| *Ixodes ricinus* | Mi5900 |  |  | 1 |  | Kammbergquelle 17 | 12/06/2021 |
| *Ixodes ricinus* | Mi5909 | 1 |  |  |  | Giebelrainquelle 16 | 11/08/2021 |
